# Effects of an 8-Week App-Based Mindfulness Intervention on Mental Health in Working Women: Randomized Controlled Trial

**DOI:** 10.2196/62814

**Published:** 2026-02-02

**Authors:** Riko Uwagawa, Koichiro Adachi, Mariko Shimoda, Ryu Takizawa

**Affiliations:** 1 Department of Clinical Psychology Graduate School of Education The University of Tokyo Tokyo Japan; 2 MRC Social, Genetic and Developmental Psychiatry Centre Institute of Psychiatry, Psychology and Neuroscience King’s College London London United Kingdom

**Keywords:** mindfulness, mobile apps, randomized controlled trial, women’s health, mental health, subjective well-being, health promotion, mHealth, application, applications, work-related stress, stress, intervention, interventions, women, mobile phone

## Abstract

**Background:**

Although working women experience increased work-related stress, preventive interventions to reduce its negative effects on their mental health are insufficient.

**Objective:**

This study evaluated the effectiveness of an 8-week mindfulness-based self-help intervention via a smartphone app across 4 domains (general psychological, work-related, family-related, and work-to-conflict) among working women.

**Methods:**

This study recruited women workers via various media sources, such as crowdsourcing sites and social networking services. Participants were randomly assigned to the intervention (n=106) or waitlist control groups (n=107). Participants in the intervention group practiced guided mindfulness meditation every day at their convenience via an app on their cell phones for 8 weeks. The app provides an 8-week program with 4 meditation contents per 2 weeks. Participants in the waitlist control group lived as usual for 8 weeks. We conducted web-based questionnaires to assess participants’ general psychological (life satisfaction, perceived stress, depressive and anxiety symptoms, trait anger, and mindfulness), work-related (work performance, job satisfaction, quantitative job overload, and job control), family-related (family satisfaction and partner satisfaction), and work-to-family conflict indicators.

**Results:**

An analysis of covariance, controlled for preintervention scores, revealed that the intervention significantly increased life satisfaction (*b*=1.47, β=0.11; *P*=.005) and decreased perceived stress (*b*=–2.00, β=–0.17; *P*=.01), depressive and anxiety symptoms (*b*=–1.24, β=–0.15; *P*=.02), and trait anger (reaction; *b*=–0.59, β=–0.11; *P*=.04). The intervention group demonstrated significantly increased life satisfaction (t_93_=–3.36; *P*=.001) and decreased depressive and anxiety symptoms (t_93_=2.35; *P*=.02).

**Conclusions:**

The app was effective in reducing perceived stress, depressive and anxiety symptoms, and trait anger (reaction), and in improving life satisfaction among working women. However, to improve work- and family-related indicators, higher-intensity interventions may be required, such as modifying the intervention content or extending its duration.

**Trial Registration:**

University Hospital Medical Information Network Clinical Trials Registry (UMIN-CTR) UMIN000051796; https://center6.umin.ac.jp/cgi-open-bin/ctr_e/ctr_view.cgi?recptno=R000059110

## Introduction

The impact of work-related stress on workers’ mental health has been recently investigated, and its significant social impact has become an issue [1]. According to the World Health Organization, work-related stress refers to “the response people may have when presented with work demands and pressures that are not matched to their knowledge and abilities and which challenge their ability to cope” [2].

Working women experience increased work-related stress compared with working men. The American Psychological Association found that women consistently exhibited higher levels of stress than men and had additional difficulty in coping [3]. Furthermore, women are more likely to develop stress-related symptoms owing to neurobiological differences, a sense of burden from the dual roles of balancing work and family, and exposure to job insecurity [4-6]. Work-family conflict of working women has a negative impact on their stress and on their physical and mental health, and a framework regarding the relationship between these is presented [7]. Work-related factors may affect women and men differently, with women possibly being further affected owing to their work and family roles. With the global aim of gender parity in the labor market [8], the number of women in the working population is expected to increase. Therefore, preventive interventions to reduce the negative effects of work-related stress on women’s mental health are required. However, such support is insufficient [9,10].

Traditionally, psychiatry has focused on the treatment of mental disorders rather than prevention. However, mental health is more than the absence of mental illness [10,11]. Therefore, interventions that focus on preventing mental health problems among women workers before they worsen and improving positive aspects, such as life satisfaction, could have positive effects on women’s well-being and their work, family, and society as a whole.

Mindfulness meditation is an effective intervention strategy for improving mental health and well-being. Mindfulness is the awareness that emerges from deliberate, nonjudgmental attention to experiences as they unfold moment-by-moment [12]. As mindfulness-based interventions reduce symptoms of depression, anxiety, and perceived stress and improve sleep quality and well-being [13-15], they are attracting attention as a preventive intervention strategy.

Additionally, the effectiveness of mindfulness meditation provided by smartphone apps has been recently highlighted. According to the International Telecommunication Union, there are over 8.89 billion mobile subscriptions worldwide [16]. Therefore, mobile technology can be used to provide preventive health care interventions to numerous people.

A traditional mindfulness-based program is high-intensity (8 weekly sessions of 2.5 hours per session and 30-40 minutes of practice per day) and time-constrained, which creates a participation barrier for nonclinical working women. The mobile-based mindfulness intervention is an app-based, voice-guided meditation practice that allows users to practice at their own convenience, which offers the advantages of high convenience and low cost [17-19]. Furthermore, online mindfulness interventions are effective in improving depression, anxiety, stress, rumination, and well-being [20,21].

However, no studies have examined the effects of mobile-based mindfulness meditation on working women from work, family, and work–family conflict aspects, as well as general measures. To our knowledge, only two studies have examined the effectiveness of mobile-based mindfulness meditation among working women. Santos et al [10] found that an app-based mindfulness and positive psychology intervention effectively reduced perceived stress and anxiety symptoms in working women. Coelhoso et al [11] revealed that a well-being mobile app designed to handle psychological stress based on relaxation training, breathing techniques, meditation (mindfulness, loving meditation, such as mindfulness, loving, kindness, and empathetic joy), and positive psychology principles improved working women’s work-related well-being and reduced their work-related and overall stress.

Conversely, no study has examined working women’s well-being from the 4 aspects of general psychological, work-related, family-related, and work-family conflict indicators. Examining their effects is essential for the future applications of mindfulness meditation, as it will help us comprehensively understand how mindfulness meditation works for women workers.

Therefore, this study aimed to evaluate the effectiveness of an 8-week mindfulness meditation intervention via a smartphone app among women workers through a randomized controlled trial (RCT). Effectiveness was examined via 4 indicators: general psychological, work-related, family-related, and work-to-conflict measures. Furthermore, we examined the measures that would effectively influence. We hypothesized that participants in the intervention group (self-care mindfulness meditation via the smartphone app) would have a higher level of general psychological (life satisfaction, perceived stress, depressive and anxiety symptoms, trait anger, and mindfulness), work-related (work performance, job satisfaction, quantitative job overload, and job control), family-related (family satisfaction and partner satisfaction), and work-to-family conflict indicators compared with those in the waitlist control group.

## Methods

### Participants

A power analysis was conducted to determine the sample size needed for this study (significance=.05; statistical power=.8; effect size=0.4), and a sample size of 100 participants per group, for a total of 200 participants, was needed. The effect size demonstrated in the meta-analysis of the effects of online mindfulness-based interventions on mental health was used as reference (depression Hedges *g*=0.34; stress Hedges *g*=0.44) [21].

This study recruited 397 women workers via various media sources, such as crowdsourcing sites and social networking services. Inclusion criteria included those who were (1) biologically female, (2) employed for at least 20 hours per week, (3) owned an iPhone (for convenience of the app used), and (4) aged 18-64 years. Exclusion criteria included those who (1) received treatment for a mental disorder, (2) scored ≥13 on the 6-item Kessler Psychological Distress Scale (K6) Japanese version, (3) were on leave, and (4) were currently pregnant or likely to become pregnant within six months. Among the participants, 95 did not meet the inclusion and exclusion criteria. Hence, 302 women workers who met the criteria were asked to respond to the preintervention assessment, and 215 who completed the assessment were randomly assigned to the intervention (n=107) or waitlist control group (n=108). Randomization was computerized using a blocked randomization scheme (block size 10). A total of 8 working women dropped out. Of the 8 participants, 2 participants (intervention group, n=1; wait-list control group, n=1) declined to participate in this study, 2 participants in the intervention group opted out of the intervention, and 4 participants (intervention group, n=2; wait-list control group, n=2) could not be contacted. After 8 weeks, the participants were asked to respond to the postintervention assessment, and 196 women workers completed the assessment (intervention group, n=95; waitlist control group, n=101). Of 215 participants who completed the preintervention assessment, 4 who worked <19 hours per week on average in the preintervention assessment were excluded from analysis (intervention group, n=1; waitlist control group, n=3). Therefore, of the 215 participants who were randomized, data from 209 participants (intervention group, n=105; waitlist control group, n=104) were finally analyzed, excluding 2 participants who declined to participate in this study and 4 participants who worked <19 hours per week on average in the preintervention assessment.

### Procedure

#### Overview

This study was designed as a parallel-design RCT. Randomization was computerized independently by research staff using a blocked randomization scheme (block size 10). Participants were expected to be randomized in a ratio of 1:1 to the intervention or waitlist control group. This study was an open-label RCT as it was not possible to blind the allocation.

This study was conducted from July 2023 to January 2024 via web forms. Participants in the intervention group installed the app for meditation after the preintervention assessment. Participants practiced guided mindfulness meditation via the app on their cell phones every day at their convenience for 8 weeks. After 8 weeks, the participants received the postintervention questionnaire via the app and email. The participants in the waitlist control group lived as usual for 8 weeks after the preintervention assessment. After 8 weeks, they also responded to the postintervention questionnaire via email.

This study was registered in the University Hospital Medical Information Network (UMIN) Clinical Trials Registry (UMIN000051796).

#### 8-Week Mindfulness-Based Self-Help Intervention via the Smartphone App

**Figure 1:**
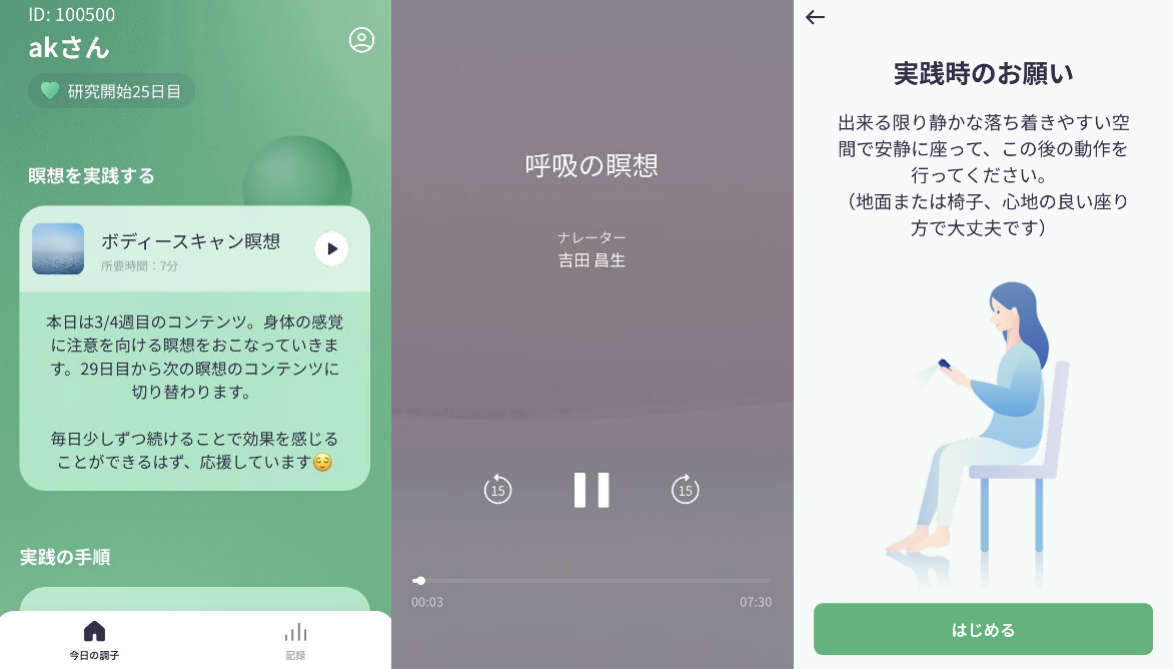
Display of the smartphone app.

**Figure 2:**
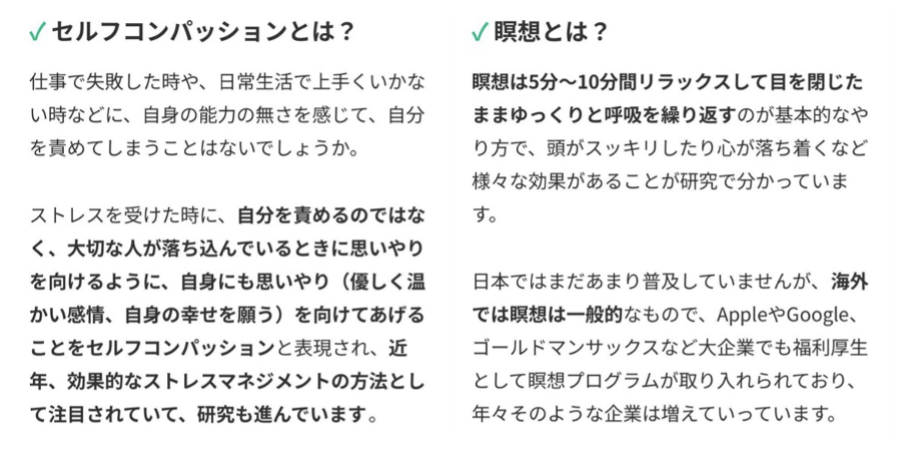
Display of the psychoeducation.

Mindfulness meditation was conducted via the iOS app, with the content changed every 2 weeks (Table 1). The app displayed the day’s meditation content and explanation on the home screen. After viewing this screen, the participants pressed the play button to hear the guided audio and practiced meditation. Figure 1 illustrates the display of the app. In addition, the psychoeducation pages on mindfulness and self-compassion were created and inserted on the app (Figure 2).

**Table 1:** Content of the 8-week self-help mindfulness-based meditation.

Week	Types of meditation	Duration (minutes)
1 and 2	Meditation of breath	7
3 and 4	Body scan	7
5 and 6	Meditation of breath, sound, and body	12
7 and 8	Loving-kindness meditation	12

The content included “meditation of breath,” “meditation of breath, sound, and body,” and “body scan meditation,” based on previous studies [22]. As the “body scan” was partially included in “meditation of breath, sound, and body,” in this study, the latter was conducted after the former. Furthermore, as the effectiveness of interventions that incorporated elements of self-compassion was recently highlighted, “loving-kindness meditation” was ultimately added. As a daily 13-minute meditation was effective after 8 weeks [23], the intervention period was designed to be 8 weeks.

### Measurements

#### General Psychological Domain: Well-Being

Well-being was assessed as life satisfaction using the 5-item Satisfaction with Life Scale. Participants evaluated their subjective life satisfaction on a 7-point Likert scale that ranged from 1 (strongly disagree) to 7 (strongly agree) [24,25]. This measurement was developed by Diener et al [24]. The development of the Japanese version used in this study and its validity and reliability were studied by Sumino [25]. Sample items included “In most ways, my life is close to my ideal.” The total score was a sum of all the individual item scores, and higher scores indicated greater life satisfaction. In this study, Cronbach α was 0.85 and 0.81 for the pre- and postintervention assessments, respectively.

#### Mental Health Outcomes

##### Perceived Stress

The 10-item Perceived Stress Scale was used to assess perceived stress. Participants rated how unpredictable, uncontrollable, and overloaded they found their lives on a 5-point Likert scale that ranged from 0 (never) to 4 (very often) [26,27]. This measurement was developed by Cohen et al [26]. The development of the Japanese version used in this study and its validity and reliability were studied by Sumi [27]. Sample items included “How often have you been upset because of something that happened unexpectedly?” The total score was a sum of the individual item scores, and higher scores indicated greater perceived stress. Cronbach α was 0.69 and 0.79 for pre- and postintervention, respectively.

##### Depressive and Anxiety Symptoms

K6 was used to assess depression and anxiety symptoms. Participants described how often they experienced depressive symptoms in the past 30 days on a 5-point Likert scale that ranged from 0 (none of the time) to 4 (all of the time) [28-30]. This measurement was developed by Kessler et al [28], and the Japanese version of it used in this study was developed by Furukawa et al [29]. The validity and reliability were studied by Furukawa et al [29] and by Sakurai et al [30]. Sample items included “How often did you feel nervous?” and “How often did you feel restless or fidgety?” The total score was a sum of all the individual item scores, and higher scores indicated a greater severity of depression and anxiety. Cronbach α was 0.83 and 0.80 for pre- and postintervention, respectively.

##### Trait Anger

“Trait anger (T-Ang; 10-item),” a subscale of the 57-item State-Trait Anger Expression Inventory 2 (STAXI-2), was used to assess the traits of anger reaction [31-33]. This measurement was developed by Spielberger [31]. The development of the Japanese version used in this study and its reliability were studied by Mine and Ohki [32] and by Mine and Sato [33]. Participants evaluated their perceptions of anger proneness on a 4-point Likert scale that ranged from 1 (strongly disagree) to 4 (strongly agree). Sample items included “I am quick-tempered.” T-Ang included two subfactors: T-Ang/Temperament (T-Ang/T; trait of feeling anger with or without stimulus) and T-Ang/Reaction (T-Ang/R; frequency of experiencing feelings of anger in situations involving irritation or negative evaluation). The total score within each subfactor and all items was calculated by summing the item scores. Higher scores indicated greater trait anger. Cronbach α for preintervention was T-Ang Cronbach α=0.84, T-Ang/T Cronbach α=0.79, and T-Ang/R Cronbach α=0.77, and for postintervention was T-Ang Cronbach α=0.83, T-Ang/T Cronbach α=0.84, and T-Ang/R Cronbach α=0.76.

##### Mindfulness

The 15-item Mindful Attention Awareness Scale was used to assess dispositional mindfulness [34,35]. This measurement was developed by Brown and Ryan [34]. The development of the Japanese version used in this study and its validity and reliability were studied by Fujino et al [35]. Participants rated the degree to which they functioned without awareness of the present experience in daily life on a 6-point scale that ranged from 1 (almost never) to 6 (almost always). Sample items included “I could be experiencing some emotion and not be conscious of it until sometime later.” All items were reversed as they assessed the lack of mindful attention and awareness. The total score was a sum of all the reversed-item scores, and higher scores indicated greater mindful attention and awareness. Cronbach α was 0.81 and 0.87 for pre- and postintervention, respectively.

### Work-Related Domain

#### Work Performance

The World Health Organization Health and Work Performance Questionnaire Short Form was used to assess work performance. The questions included: “On a scale of 0-10, where 0 is the worst job performance anyone could have at your job, and 10 is the performance of a top worker, how would you rate the usual performance of most workers in a job similar to yours?” (possible performance) and “Using the same 0-10 scale, how would you rate your overall job performance on the days you worked during the past four weeks?”(actual performance) [36-38]. This measurement was developed by Kessler et al [36]. The development of the Japanese version used in this study and its validity and reliability were studied by Kawakami et al [38]. Participants evaluated the workplace costs of health problems regarding self-reported sickness leaves and reduced job performance (presenteeism). Presenteeism was assessed by “absolute” and “relative presenteeism.” “Absolute presenteeism” was calculated by multiplying the score of actual performance by 10. Higher scores indicated greater performance. “Relative presenteeism” was calculated by the ratio of actual performance to possible performance (restricted to the range of 0.25–2.0, where values <0.25 and >2.0 were converted to 0.25 and 2.0, respectively). Higher scores indicated greater performance.

#### Job Satisfaction

Job satisfaction was assessed via a single item from the Brief Job Stress Questionnaire (BJSQ) [39]. The development of this measurement used in this study and its validity and reliability were studied by Inoue et al [39]. Participants rated the degree to which they agreed with the item, “I am satisfied with my job,” on a 4-point Likert scale that ranged from 1 (satisfied) to 4 (dissatisfied). The item was reversed as it assessed the high level of job satisfaction. Higher scores indicated greater job satisfaction.

#### Quantitative Job Overload

“Quantitative job overload (3-item),” a subscale of the BJSQ, was used to assess job overload [39]. Participants rated the degree of their job overload on a 4-point Likert scale that ranged from 1 (agree) to 4 (disagree). Sample items included “I have a lot of work to do.” All items were reversed as they assessed the high level of job overload. The total score was a sum of all the reversed-item scores, and higher scores indicated a greater job overload. Cronbach α was 0.64 and 0.56 for pre- and postintervention, respectively.

#### Job Control

“Job control (3-item),” a subscale of the BJSQ, was used to assess job control [39]. Participants rated the degree of their job control on a 4-point Likert scale that ranged from 1 (agree) to 4 (disagree). Sample items included “I can work at my own pace.” All items were reversed as they assessed the high level of job control. The total score was a sum of all the reversed-item scores, and higher scores indicated a greater sense of job control. Cronbach α was 0.60 and 0.64 for pre- and postintervention, respectively.

### Family-Related Domain

#### Family Satisfaction

Family satisfaction was assessed via a single item from the BJSQ [39]. Participants rated the degree to which they agreed with the item, “I am satisfied with my family life,” on a 4-point Likert scale that ranged from 1 (satisfied) to 4 (dissatisfied). The item was reversed as it assessed the high level of family satisfaction. Higher scores indicated greater family satisfaction.

#### Partner Satisfaction

Partner satisfaction was assessed via a single item: “Using the 10-point scale, how would you rate your current level of satisfaction with your relationship with your partner?” Only participants who lived with their partners were asked to respond. Participants rated the degree of their satisfaction with their partner on a 10-point Likert scale that ranged from 1 (dissatisfied) to 10 (satisfied). Higher scores indicated greater satisfaction.

#### Work-to-Family Conflict Domain

The 22-item Survey Work-Home Interaction-Nijmegen was used to assess the 4 subscales that reflected the underlying dimensions of work–family spillover: (1) work-family negative spillover (WFNS, 8 items; eg, “You do not have the energy to engage in leisure activities with your spouse/family/friends because of your job.”), (2) family-work negative spillover (FWNS, 4 items; eg, “You do not feel like working because of problems with your spouse/family/friends.”), (3) work-family positive spillover (WFPS, 5 items; eg, “You fulfill your domestic obligations better because of the things you have learned on your job.”), (4) family-work positive spillover (FWPS, 5 items; eg, “You have greater self-confidence at work because you have your home life well organized”) [40,41]. This measurement was developed by Geurts et al [40] in 2005. The development of the Japanese version used in this study and its validity and reliability were studied by Shimada et al [41]. Responses were rated on a 4-point Likert scale that ranged from 0 (never) to 3 (always). The total score of each subscale was calculated as a sum of all the individual item scores. Higher scores on the positive (WFPS and FWPS) and negative spillover subscales (WFNS and FWNS) indicated greater positive and negative impacts, respectively. For preintervention, the Cronbach α were WFNS Cronbach α=0.88, FWNS Cronbach α=0.79, WFPS Cronbach α=0.73, and FWPS Cronbach α=0.78, and for postintervention, it was WFNS Cronbach α=0.89, FWNS Cronbach α=0.76, WFPS Cronbach α=0.79, and FWPS Cronbach α=0.83.

### Statistical Analysis

We conducted Chi-squared, *t* tests, and the Fisher exact test in order to examine whether there are differences in demographic variables and psychological indices between the intervention and control groups. Subsequently, we conducted 2-tailed *t* tests to examine whether there were differences in demographic variables and psychological indices of participants in the intervention and waitlist control groups, respectively.

For the intervention effects, we conducted an analysis of covariance (ANCOVA; independent variables: intervention group=1 and waitlist control group=0) that used the least squares method as an estimation method, controlled for preintervention scores. We conducted an ANCOVA that used the least squares estimation method, controlled for preintervention scores, age, employment status (regular employment: employed full time with no fixed term of employment; nonregular employment: not regular employment), psychiatric history, education, and marital status. In this study, the participants were randomly assigned to the intervention and control groups. However, because of the possibility that the intervention effect might not be properly detected due to group differences in preintervention scores and demographic data, we controlled for them. Additionally, paired *t* tests were conducted to determine any differences in the pre- and postintervention assessments within each group. An intention-to-treatment analysis was used. R (version 4.3.2; R Foundation for Statistical Computing) was used for statistical analysis.

### Ethical Considerations

This study was approved by the Life Science Research Ethics and Safety Committee, the University of Tokyo (23-144, 23-227, and 24-020).

## Results

### Baseline

**Figure 3:**
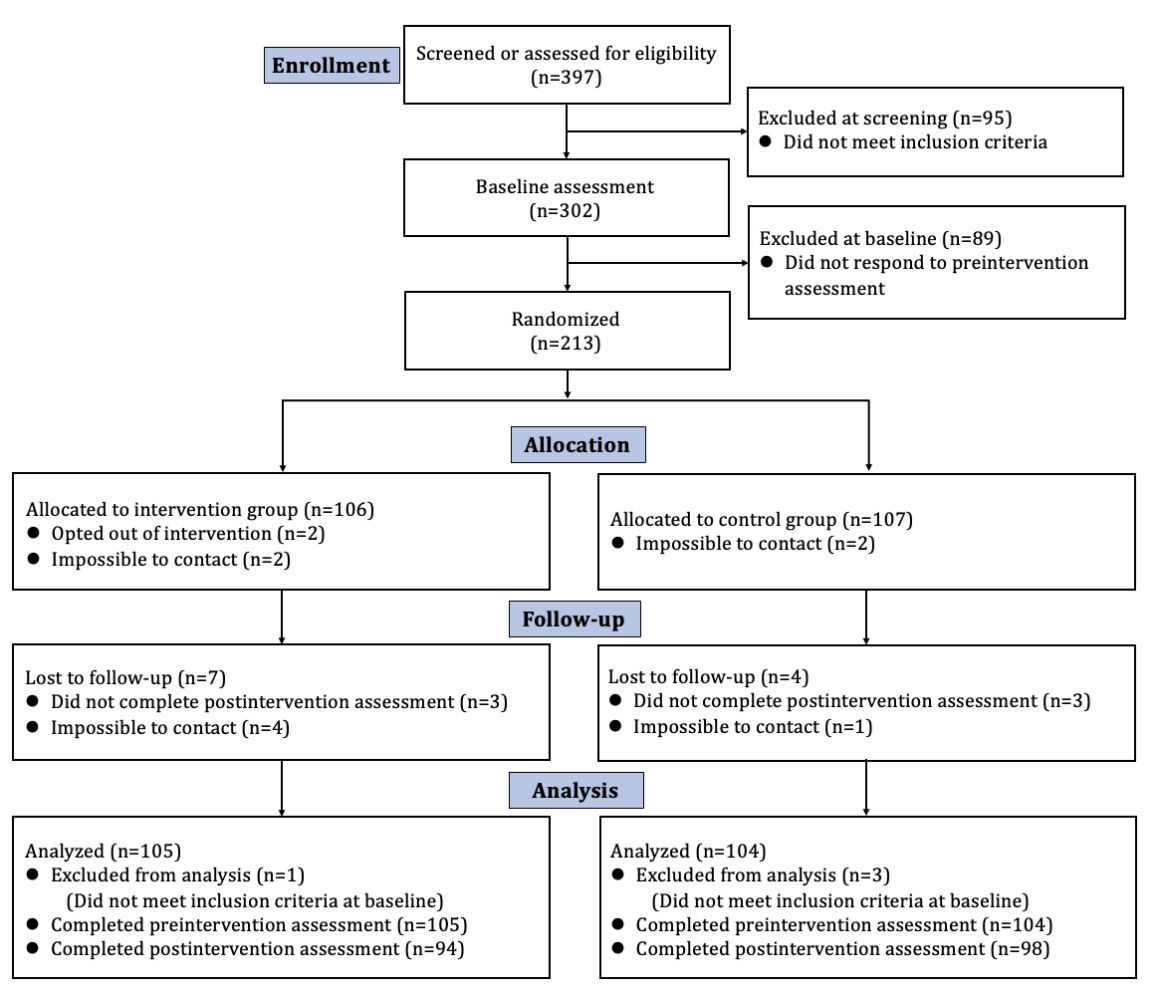
CONSORT (Consolidated Standards for Reporting Trials) flowchart for participants.

Figure 3 illustrates the CONSORT (Consolidated Standards for Reporting Trials) flow diagram (the CONSORT checklist is provided in Multimedia Appendix 1).

Table 2 shows the participants’ demographic characteristics. Chi-squared and *t* tests revealed no differences in demographic variables and psychological indices between the intervention and waitlist control groups (*P*>.05).

**Table 2:** Participants’ demographic information.

Participant characteristics			Intervention group (n*=*105)		Waitlist control group (n=104)		Difference statistic: t test (*df*) or chi-square (*df*)		*P* value
Age (years), mean (SD)			36.81 (10.82)		36.81 (10.70)		0.0 (207)^a^		.99
**Education level,** **n** **(%)**							Fisher exact test		.09
	Less than a bachelor’s degree	46 (43.6)		31 (29.8)					
	Bachelor’s degree	48 (45.7)		60 (57.7)					
	Master’s degree	11 (10.6)		11 (10.6)					
	Doctoral degree	0 (0)		2 (1.9)					
**Marital status,** **n** **(%)**							Fisher exact test		.44
	Married	46 (46.8)		50 (48.1)					
	Single	51 (44.7)		42 (40.4)					
	Divorced	6 (6.4)		11 (10.6)					
	Widowed	2 (2.1)		1 (1)					
**Employment status^b^, n (%)**							2.1 (1)^c^		.14
	Regular employment	66 (62.9)		54 (51.9)					
	Nonregular employment	39 (37.1)		50 (48.1)					
**Psychiatric history^d^, n (%)**							2.6 (1)^c^		.11
	Yes	7 (6.7)		15 (14.4)					
	No	98 (93.3)		89 (85.6)					
**Living with a partner, n (%)**							0.0 (1)^c^		.83
	Yes	52 (49.5)		54 (51.9)					
	No	53 (50.5)		50 (48.1)					
**Youngest child age in years, n (%)**							2.1 (3)^c^		.56
	0-2	8 (7.6)		5 (4.8)					
	3-18	30 (28.6)		30 (28.8)					
	19+	4 (3.8)		8 (7.7)					
	None	63 (6)		61 (58.7)					

^a^*t* test (*df*).

^b^Employment status indicates whether the individual is a regular employee.

^c^Chi-square (*df*).

^d^Psychiatric history indicates whether the individual has a history of visiting a psychosomatic medicine or psychiatric clinic.

### Comparing Completers and Dropouts Within Each Group

In the intervention group, no statistically significant differences were observed in demographic information and psychological measurement scores between the dropouts and participants who completed the postintervention assessment (*P*>.05). In the waitlist control group, there were differences in age (t_102_=2.66; *P*=.009), T-Ang (t_102_=–2.22; Cohen *d*=0.93; *P*=.03), T-Ang/R (t_102_=–2.26; Cohen *d*=0.95; *P*=.03), and job control (t_102_=–2.57; Cohen *d*=1.08; *P*=.01). Dropouts were significantly younger (mean 25.83, SD 4.62), more angry (T-Ang: mean 24.00, SD 9.06; T-Ang/R: mean 11.00, SD 4.65), and perceived an additional sense of job control (mean 9.17, SD 1.60) compared with the retained participants (age mean 37.48, SD 10.61; T-Ang: mean 18.76, SD 5.39; T-Ang/R: mean 8.34, SD 2.68; job control: mean 6.56, SD 2.45).

### Practice Frequency

Participants in the intervention group used the app for a mean of 42.32 days (75.57%, SD 15.63) in 8 weeks.

### Outcomes

#### Group Effects

Table 3 presents the scores of the pre- and postintervention assessments. Table 4 presents the results of ANCOVA. The ANCOVA, controlled for preintervention scores, revealed significant group effects on life satisfaction (*b*=1.47, β=0.11; *P*=.005), perceived stress (*b*=–2.00, β=–0.17; *P*=.01), depressive and anxiety symptoms (*b*=–1.24, β=–0.15; *P*=.02), and T-Ang/R (*b*=–0.59, β=–0.11; *P*=.04). The ANCOVA, controlled for pre-intervention scores and demographic data (age, employment status, psychiatric history, education, marital status), revealed significant group effects on life satisfaction (*b*=1.35, β=0.10; *P*=.02), perceived stress (*b*=–1.91, β=–0.16; *P*=.02), depressive and anxiety symptoms (*b*=–1.13, β=–0.13; *P*=.03), and T-Ang/R (*b*=–0.71, β=–0.13; *P*=.02).

**Table 3:** Scores of the pre- and postintervention assessments.

				Intervention group												Waitlist control group												*t* test						
				Mean (SD)					*P* value			Cohen *d* (95% Cl)			Mean (SD)						*P* value			Cohen *d* (95% Cl)			*P* value				Cohen *d* (95% Cl)			
				Pre		Post								Pre				Post																
**General psychological domain**																																		
	Life satisfaction		17.70 (6.68)		18.80 (6.76)		.001			0.22 (0.09 to 0.35)			18.62 (6.89)				18.58 (6.32)		.38			0.05 (–0.06 to 0.15)			.82				0.03 (–0.25 to 0.31)					
	Perceived stress		18.71 (5.14)		18.65 (5.65)		.67			0.05 (–0.19 to 0.29)			17.62 (5.95)				19.94 (6.38)		<.001			0.40 (0.21 to 0.60)			.14				0.21 (–0.07 to 0.50)					
	Depressive and anxiety symptoms		6.16 (4.06)		5.37 (3.61)		.02			0.27 (0.04 to 0.50)			6.20 (4.89)				6.41 (4.71)		.33			0.08 (–0.08 to 0.25)			.09				0.25 (–0.04 to 0.53)					
	**Trait anger**		18.50 (5.16)		18.27 (5.31)		.17			0.10 (–0.04 to 0.23)			19.06 (5.73)				18.92 (5.58)		.70			0.03 (–0.12 to 0.18)			.41				0.12 (–0.17 to 0.40)					
		Trait anger (temperament)	7.09 (2.62)		6.94 (2.54)		.06			0.12 (–0.01 to 0.24)			7.49 (2.78)				7.30 (2.95)		.56			0.04 (–0.10 to 0.19)			.37				0.13 (–0.15 to 0.41)					
		Trait anger (reaction)	8.50 (2.65)		8.28 (2.71)		.24			0.10 (–0.07 to 0.27)			8.49 (2.86)				8.71 (2.74)		.07			0.14 (–0.01 to 0.29)			.27				0.16 (–0.12 to 0.44)					
	Mindfulness		43.36 (10.65)		44.05 (11.31)		.70			0.03 (–0.10 to 0.07)			43.74 (9.85)				44.33 (11.75)		.72			0.02 (–0.11 to 0.16)			.87				0.02 (–0.26 to 0.31)					
**Work**																																		
	**Work performance**																																	
		Absolute presenteeism	61.43 (19.24)		62.02 (18.64)		.73			0.04 (–0.19 to 0.27)			61.06 (19.5)				61.84 (18.69)		.67			0.05 (–0.17 to 0.27)			.95				0.01 (–0.27 to 0.29)					
		Relative presenteeism	1.02 (0.32)		1.05 (0.27)		.50			0.09 (–0.17 to 0.34)			1.00 (0.33)				1.01 (0.32)		.67			0.05 (–0.19 to 0.30)			.35				0.14 (–0.15 to 0.42)					
	Job satisfaction		2.70 (0.72)		2.67 (0.79)		.90			0.01 (–0.21 to 0.24)			2.81 (0.87)				2.81 (0.83)		.53			0.05 (–0.10 to 0.20)			.25				0.17 (–0.12 to 0.45)					
	Quantitative job overload		8.10 (2.36)		8.11 (2.43)		.82			0.02 (–0.17 to 0.22)			7.68 (2.59)				7.85 (2.30)		.43			0.07 (–0.10 to 0.23)			.45				0.11 (–0.17 to 0.39)					
	Job control		8.36 (2.33)		8.54 (2.23)		.17			0.11 (–0.05 to 0.26)			8.29 (2.48)				8.40 (2.29)		.84			0.02 (–0.15 to 0.18)			.66				0.06 (–0.22 to 0.35)					
**Family**																																		
	Family satisfaction		3.00 (0.77)		3.06 (0.81)		.18			0.13 (–0.06 to 0.33)			2.99 (0.82)				2.96 (0.88)		.45			0.07 (–0.12 to 0.26)			.40				0.12 (–0.16 to 0.41)					
	Partner satisfaction		7.52 (2.14)		7.39 (2.25)		.82			0.02 (–0.15 to 0.19)			7.69 (2.05)				7.15 (2.43)		.02			0.25 (0.04 to 0.45)			.62				0.10 (–0.29 to 0.49)					
**Work-to-family conflict**																																		
	Work-family negative spillover		5.62 (4.92)		5.63 (4.48)		.84			0.02 (–0.14 to 0.17)			5.51 (5.51)				5.18 (5.23)		.20			0.08 (–0.04 to 0.20)			.53				0.09 (–0.19 to 0.37)					
	Family-work negative spillover		1.25 (1.71)		1.20 (1.72)		.64			0.05 (–0.16 to 0.26)			1.19 (1.66)				1.36 (1.85)		.33			0.09 (–0.09 to 0.28)			.55				0.09 (–0.20 to 0.37)					
	Work-family positive spillover		7.08 (3.04)		7.02 (3.05)		.92			0.01 (–0.18 to 0.20)			6.77 (3.30)				7.13 (3.72)		.34			0.08 (–0.09 to 0.25)			.82				0.03 (–0.25 to 0.32)					
	Family-work positive spillover		7.09 (3.51)		7.22 (3.55)		.66			0.04 (–0.13 to 0.21)			7.19 (3.94)				7.28 (4.06)		.98			0.00 (–0.17 to 0.16)			.92				0.01 (–0.27 to 0.30)					

**Table 4:** Comparison between the control and the intervention groups.

			Controlling for prescores						Controlling for prescores and demographic data				
			*b*	β	SE	*t* test (*df*)	*P* value	*b*		β	SE	*t* test (*df*)	*P* value
**General psychological domain**													
	Life satisfaction		1.47	0.11	0.52	2.82 (188)	.005	1.35		0.10	0.55	2.47 (181)	.02
	Perceived stress		–2.00	–0.17	0.79	–2.55 (188)	.01	–1.91		–0.16	0.82	–2.34 (181)	.02
	Depressive and anxiety symptoms		–1.24	–0.15	0.51	–2.43 (188)	.02	–1.13		–0.13	0.53	–2.14 (181)	.03
	**Trait anger**		–0.66	–0.06	0.53	–1.26 (188)	.21	–0.88		–0.08	0.54	–1.64 (181)	.10
		Trait anger (temperament)	–0.22	–0.04	0.25	–0.85 (188)	.40	–0.23		–0.04	0.26	–0.89 (181)	.37
		Trait anger (reaction)	–0.59	–0.11	0.29	–2.03 (188)	.04	–0.71		–0.13	0.29	–2.41 (181)	.02
	Mindfulness		–0.02	0.00	1.03	–0.01 (188)	.99	0.11		0.00	1.08	0.10 (181)	.92
**Work**													
	**Work performance**												
		Absolute presenteeism	0.05	0.00	2.49	0.02 (188)	.98	–0.33		–0.01	2.60	–0.13 (181)	.90
		Relative presenteeism	0.03	0.06	0.04	0.80 (188)	.42	0.02		0.04	0.04	0.53 (181)	.60
	Job satisfaction		–0.03	–0.02	0.10	–0.26 (188)	.79	–0.01		0.00	0.10	–0.07 (181)	.94
	Quantitative job overload		–0.01	0.00	0.28	–0.05 (188)	.96	0.00		0.00	0.29	0.01 (181)	.99
	Job control		0.24	0.05	0.24	0.99 (188)	.32	0.25		0.06	0.24	1.03 (181)	.30
**Family**													
	Family satisfaction		0.14	0.08	0.10	1.37 (188)	.17	0.14		0.08	0.10	1.32 (181)	.19
	Partner satisfaction		0.50	0.11	0.30	1.64 (188)	.10	0.38		0.08	0.32	1.20 (181)	.23
**Work-to-family conflict**													
	Work-family negative spillover		0.37	0.04	0.44	0.84 (188)	.40	0.28		0.03	0.46	0.60 (181)	.55
	Family-work negative spillover		–0.21	–0.06	0.22	–0.93 (188)	.35	–0.23		–0.06	0.23	–1.00 (181)	.32
	Work-family positive spillover		–0.21	–0.03	0.40	–0.53 (188)	.60	–0.06		–0.01	0.41	–0.15 (181)	.88
	Family-work positive spillover		0.07	0.01	0.42	0.16 (188)	.87	–0.04		0.00	0.43	–0.09 (181)	.93

#### Differences Between Pre- and Postintervention Assessment Within Each Group

Regarding the intervention group, the postintervention scores of life satisfaction were significantly higher (mean_pre_ 17.70, SD_pre_ 6.68; mean_post_ 18.80, SD_post_ 6.76; t_93_=–3.36; Cohen *d*=0.22, 95% CI 0.09-0.35; *P*=.001) and those of depressive and anxiety symptoms were significantly lower (mean_pre_ 6.16, SD_pre_ 4.06; mean_post_ 5.37, SD_post_ 3.61; t_93_=2.35; Cohen *d*=0.27, 95% CI 0.04-0.50; *P*=.02) than the preintervention scores.

Regarding the waitlist control group, the postintervention scores of perceived stress were significantly higher (mean_pre_ 17.62, SD_pre_ 5.95; mean_post_ 19.94, SD_post_ 6.38; t_97_=–4.20; Cohen *d*=0.40, 95% CI 0.21-0.60; *P*<.001) and those of partner satisfaction were significantly lower than the preintervention scores (mean_pre_ 7.69, SD_pre_ 2.05; mean_post_ 7.15, SD_post_ 2.43; t_50_=2.41; Cohen *d*=0.25, 95% CI 0.04-0.45; *P*=.02).

## Discussion

### Principal Findings

This study examined the effectiveness of a mindfulness meditation intervention via a smartphone app among healthy women workers. To our knowledge, this was the first study that examined the effects of the mindfulness meditation intervention via a smartphone app on 4 domains (psychological, work, family, and work-to-family conflict) among women workers. Women workers who received the intervention demonstrated higher postintervention scores on the general psychological indicators (life satisfaction, perceived stress, depressive and anxiety symptoms, and trait anger (reaction) than those in the waitlist control group, controlled for preintervention scores as well as age, employment status, psychiatric history, education, and marital status. However, the intervention was not effective in the other 3 domains (work, family, and work-to-family conflict). In particular, life satisfaction and depression, and anxiety symptoms significantly improved in the intervention group.

Our results corroborated the findings of Santos et al [10] and Coelhoso et al [11] that app-based mindfulness interventions reduced perceived stress and anxiety symptoms and improved subjective well-being among working women. Additionally, we found that app-based mindfulness interventions were useful for reducing reactive anger in working women. Working women are more likely to experience stress owing to neurobiological differences and balancing work and family than men, which may impair their well-being [3-7]. Mindfulness interventions enhance acceptance and observation skills by halting in daily life, paying attention to what is happening “here and now,” and observing and accepting things as they are [12,42,43]. Therefore, acceptance and observation skills enable working women to pause and look at things as they are without being overwhelmed by negative thoughts and feelings when they are burdened by work and family in their daily lives. This is likely to calm their anger, lower their subjective stress, and increase their sense of well-being.

Conversely, this study observed no improvements in work-related, family-related, and work-to-family conflict indicators after the intervention. Previous studies have reported that mindfulness interventions increase family satisfaction among elementary and secondary school teachers, partner satisfaction among participants in a romantic relationship, and work satisfaction and performance among workers, and decrease the work-to-family conflict among workers [44-50]. The inconsistency of our results with those of previous studies could be owing to differences in sex, intervention duration, and meditation time per session. Previous studies examining the effects of preventive online mindfulness interventions for nonclinical populations on perceived stress and mindfulness have shown substantial differences across studies regarding design, setting, participants’ age, gender ratio, intervention characteristics, and outcome measures [51]. These factors have been suggested to potentially influence the magnitude of the observed effects. Moreover, this preventive intervention may have been too short to reduce burden in the 3 work- or family-related domains. Furthermore, the intervention content was aimed at general meditation (“mindfulness of breath,” “body scan,” “mindfulness of breath, sound, and body,” and “loving-kindness meditation”), rather than work- or family-specific content, and was implemented in a specific order. Therefore, the 8-week low-intensity meditation intervention could have led to an improvement in the individual’s general well-being; however, the effect on work- or family-related indicators may have occurred after a few months. Alternatively, higher-intensity interventions may be required, such as modifying the intervention’s contents or extending its duration. Additionally, only 1 item was used to measure work performance and job, family, and partner satisfaction, whereas 3 items were used to measure job overload and control. Therefore, the number of questions may have been too small to detect significant differences.

### Limitations and Future Directions

This study has some limitations: a lack of subgroup analysis, an intervention not designed specifically for the target or context, and problems with generalizability and variability in the intensity of the intervention due to the application and problems with the scales used. First, in this study, subgroup analyses were not conducted to examine the impact of the subjects’ traits on intervention effects. The effects of our mindfulness intervention on general psychological, work-related, family-related, and work-to-family conflict indicators may differ based on other factors. Some participants may have benefited from work-related, family-related, or work-to-conflict indicators. Therefore, it is necessary to examine the factors that moderate the effect of mindfulness interventions.

Second, the mindfulness intervention used in this study was not designed as target- and context-specific. Previous studies have developed target- and context-specific mindfulness interventions, such as for the workplace and parenting. Therefore, future studies should be designed specifically for working women, with an aim to increase the effects on work- and family-related indicators.

Third, there are two limitations of using an app for the intervention: the quality of the intervention cannot be assessed, and generalizability is limited due to restrictions on participant conditions. Since a self-help app was used as the intervention in this study, it was not possible to assess how well participants were focused on meditation, which may have resulted in variability in the effectiveness of the intervention. In addition, the limitations of the app used for the intervention limited the participants in this study to iPhone users, which may have biased the sample and limited generalizability. Therefore, future studies should address compliance issues to address these limitations caused by the app without limiting participants to iPhone users.

Fourth, some of the scales used had few items. The small number of items might have prevented the detection of significant differences. Therefore, future research should increase the number of items used in the survey.

### Conclusion

This study examined the effects of mindfulness interventions via a smartphone app on women workers’ general psychological, work-related, family-related, and work-to-family conflict indicators through an RCT. Our results revealed that the intervention increased life satisfaction and reduced perceived stress, depressive and anxiety symptoms, and anger reactions.

## Appendix

Multimedia Appendix 1 CONSORT-EHEALTH checklist (V 1.6.1).

## Abbreviations

ANCOVA: analysis of covariance
BJSQ: Brief Job Stress Questionnaire
CONSORT: Consolidated Standards for Reporting Trials
FWNS: family-work negative spillover
FWPS: family-work positive spillover
K6: 6-item Kessler Psychological Distress Scale
RCT: randomized controlled trial
STAXI-2: State-Trait Anger Expression Inventory 2
T-Ang/R: Trait-anger/reaction
T-Ang/T: Trait-anger/temperament
UMIN: University Hospital Medical Information Network
WFNS: work-family negative spillover
WFPS: work-family positive spillover

## References

[1] Hassard J, Teoh KRH, Visockaite G, Dewe P, Cox T (2018). The cost of work-related stress to society: a systematic review. J Occup Health Psychol.

[2] (2020). Occupational health: stress at the workplace. World Health Organization.

[3] (2023). Stress in America 2023. A nation recovering from collective trauma. American Psychological Association.

[4] Kawakami N, Haratani T, Kobayashi F, Ishizaki M, Hayashi T, Fujita O, Aizawa Y, Miyazaki S, Hiro H, Masumoto T, Hashimoto S, Araki S (2004). Occupational class and exposure to job stressors among employed men and women in Japan. J Epidemiol.

[5] Savic I, Perski A, Osika W (2018). MRI shows that exhaustion syndrome due to chronic occupational stress is associated with partially reversible cerebral changes. Cereb Cortex.

[6] Peristera P, Westerlund H, Magnusson Hanson LL (2018). Paid and unpaid working hours among Swedish men and women in relation to depressive symptom trajectories: results from four waves of the Swedish longitudinal occupational survey of health. BMJ Open.

[7] Poms L, Fleming L, Jacobsen K (2016). Work-family conflict, stress, and physical and mental health: a model for understanding barriers to and opportunities for women's well-being at home and in the workplace. World Med Health Policy.

[8] (2023). Global gender gap report 2023. World Economic Forum.

[9] Ornek OK, Esin MN (2020). Effects of a work-related stress model based mental health promotion program on job stress, stress reactions and coping profiles of women workers: a control groups study. BMC Public Health.

[10] Santos FRMD, Lacerda SS, Coelhoso CC, Barrichello CR, Tobo PR, Kozasa EH (2021). The integration of meditation and positive psychology practices to relieve stress in women workers (Flourish): effects in two pilot studies. Behav Sci (Basel).

[11] Coelhoso CC, Tobo PR, Lacerda SS, Lima AH, Barrichello CRC, Amaro E, et al (2019). A new mental health mobile app for well-being and stress reduction in working women: randomized controlled trial. J Med Internet Res.

[12] Kabat-Zinn J (2003). Mindfulness-based interventions in context: past, present, and future. Clinical Psychology: Science and Practice.

[13] Khoury B, Sharma M, Rush SE, Fournier C (2015). Mindfulness-based stress reduction for healthy individuals: a meta-analysis. J Psychosom Res.

[14] Galante J, Friedrich C, Dawson AF, Modrego-Alarcón M, Gebbing P, Delgado-Suárez I, et al (2021). Mindfulness-based programmes for mental health promotion in adults in nonclinical settings: a systematic review and meta-analysis of randomised controlled trials. PLoS Med.

[15] Rusch H, Rosario M, Levison L, Olivera A, Livingston WS, Wu T, et al (2019). The effect of mindfulness meditation on sleep quality: a systematic review and meta-analysis of randomized controlled trials. Ann N Y Acad Sci.

[16] (2024). Key ICT indicators for the world and special regions (totals and penetration rates). The International Telecommunication Union.

[17] Plaza I, Demarzo MMP, Herrera-Mercadal P, García-Campayo J (2013). Mindfulness-based mobile applications: literature review and analysis of current features. JMIR Mhealth Uhealth.

[18] Flynn S, Hastings RP, Burke C, Howes S, Lunsky Y, Weiss JA, Bailey T (2020). Online mindfulness stress intervention for family carers of children and adults with intellectual disabilities: feasibility randomized controlled trial. Mindfulness.

[19] Liu C, Chen H, Zhou F, Long Q, Wu K, Lo L, et al (2022). Positive intervention effect of mobile health application based on mindfulness and social support theory on postpartum depression symptoms of puerperae. BMC Womens Health.

[20] Rigabert A, Motrico E, Moreno-Peral P, Resurrección DM, Conejo-Cerón S, Cuijpers P, et al (2020). Effectiveness of online psychological and psychoeducational interventions to prevent depression: Systematic review and meta-analysis of randomized controlled trials. Clin Psychol Rev.

[21] Sommers-Spijkerman M, Austin J, Bohlmeijer E, Pots W (2021). New Evidence in the booming field of online mindfulness: an updated meta-analysis of randomized controlled trials. JMIR Ment Health.

[22] Armstrong L (2012). An investigation into the effects of a short-term mindfulness intervention on stress and emotion regulation in undergraduate students: understanding mechanisms of action. Manchester Metropolitan University.

[23] Basso JC, McHale A, Ende V, Oberlin DJ, Suzuki WA (2019). Brief, daily meditation enhances attention, memory, mood, and emotional regulation in non-experienced meditators. Behav Brain Res.

[24] Diener E, Emmons RA, Larsen RJ, Griffin S (1985). The satisfaction with life scale. J Pers Assess.

[25] Sumino Z (1994). Development of the Japanese version of the satisfaction with life scale.

[26] Cohen S, Kamarck T, Mermelstein R (1983). A global measure of perceived stress. J Health Soc Behav.

[27] Sumi K (2006). Reliability and validity of the Japanese version of the perceived stress scale [Article in Japanese]. Jpn J Health Psychol.

[28] Kessler RC, Andrews G, Colpe LJ, Hiripi E, Mroczek DK, Normand SLT, et al (2002). Short screening scales to monitor population prevalences and trends in non-specific psychological distress. Psychol Med.

[29] Furukawa TA, Kawakami N, Saitoh M, Ono Y, Nakane Y, Nakamura Y, et al (2008). The performance of the Japanese version of the K6 and K10 in the world mental health survey Japan. Int J Methods Psychiatr Res.

[30] Sakurai K, Nishi A, Kondo K, Yanagida K, Kawakami N (2011). Screening performance of K6/K10 and other screening instruments for mood and anxiety disorders in Japan. Psychiatry Clin Neurosci.

[31] Spielberger CD (1999). State-Trait Anger Expression Inventory (Staxi): Professional Manual.

[32] Mine H, Ohki M (2001). Applicability of anger expression inventory (STAXI-2) to Japanese.

[33] Mine H, Sato S (2005). An attempt for the assessment of anger expression behavior (STAXI-2)--- comparison of anger expression behavior with U.S. and Japan ---.

[34] Brown KW, Ryan RM (2003). The benefits of being present: mindfulness and its role in psychological well-being. J Pers Soc Psychol.

[35] Fujino M, Kajimura S, Nomura M (2015). Development and validation of the Japanese version of the mindful attention awareness scale using item response theory analysis [Article in Japanese]. Jpn J Personal.

[36] Kessler RC, Barber C, Beck A, Berglund P, Cleary PD, McKenas D, Pronk N, Simon G, Stang P, Ustun TB, Wang P (2003). The world health organization health and work performance questionnaire (HPQ). J Occup Environ Med.

[37] Kessler R, Ames M, Hymel P, Loeppke R, McKenas DK, Richling DE, et al (2004). Using the world health organization health and work performance questionnaire (HPQ) to evaluate the indirect workplace costs of illness. J Occup Environ Med.

[38] Kawakami N, Inoue A, Tsuchiya M, Watanabe K, Imamura K, Iida M, et al (2020). Construct validity and test-retest reliability of the world mental health japan version of the world health organization health and work performance questionnaire short version: a preliminary study. Ind Health.

[39] Inoue A, Kawakami N, Shimomitsu T, Tsutsumi A, Haratani T, Yoshikawa T, et al (2014). Development of a short version of the new brief job stress questionnaire. Ind Health.

[40] Geurts SAE, Taris TW, Kompier MAJ, Dikkers JSE, Van Hooff MLM, Kinnunen UM (2005). Work-home interaction from a work psychological perspective: development and validation of a new questionnaire, the SWING. Work & Stress.

[41] Shimada K, Shimazu A, Geurts SAE, Kawakami N (2018). Reliability and validity of the Japanese version of the survey work–home interaction – NijmeGen, the SWING (SWING-J). Community, Work & Family.

[42] Siegel RD, Germer CK, Olendzki A (2009). Mindfulness: What is it? Where did it come from?. Clinical Handbook of Mindfulness.

[43] Hölzel BK, Lazar SW, Gard T, Schuman-Olivier Z, Vago DR, Ott U (2011). How does mindfulness meditation work? Proposing mechanisms of action from a conceptual and neural perspective. Perspect Psychol Sci.

[44] Crain TL, Schonert-Reichl KA, Roeser RW (2017). Cultivating teacher mindfulness: effects of a randomized controlled trial on work, home, and sleep outcomes. J Occup Health Psychol.

[45] Espel-Huynh H, Baldwin M, Puzia M, Huberty J (2022). The indirect effects of a mindfulness mobile app on productivity through changes in sleep among retail employees: secondary analysis. JMIR Mhealth Uhealth.

[46] Hülsheger UR, Alberts HJEM, Feinholdt A, Lang JWB (2013). Benefits of mindfulness at work: the role of mindfulness in emotion regulation, emotional exhaustion, and job satisfaction. J Appl Psychol.

[47] Kappen G, Karremans JC, Burk WJ (2019). Effects of a short online mindfulness intervention on relationship satisfaction and partner acceptance: the moderating role of trait mindfulness. Mindfulness.

[48] Kiburz KM, Allen TD, French KA (2017). Work–family conflict and mindfulness: investigating the effectiveness of a brief training intervention. J Organ Behavior.

[49] Nicklin JM, Shockley KM, Dodd H (2022). Self-compassion: implications for work-family conflict and balance. J Vocat Behav.

[50] Slutsky J, Chin B, Raye J, Creswell JD (2019). Mindfulness training improves employee well-being: a randomized controlled trial. J Occup Health Psychol.

[51] Jayewardene WP, Lohrmann DK, Erbe RG, Torabi MR (2017). Effects of preventive online mindfulness interventions on stress and mindfulness: a meta-analysis of randomized controlled trials. Prev Med Rep.

