# Supplementary material for: Effects of an 8-Week App-Based Mindfulness Intervention on Mental Health in Working Women: Randomized Controlled Trial
Source: J Med Internet Res. 2026 Feb 2;28:e62814. doi: 10.2196/62814 (PMC12910267; doi:10.2196/62814)
Supplement: Multimedia Appendix 1 [file jmir_v28i1e62814_app1.pdf]

CONSORT-EHEALTH (V 1.6.1) - Submission/Publication Form

The CONSORT-EHEALTH checklist is intended for authors of randomized trials evaluating web-based and Internet-based applications/interventions, including mobile interventions, electronic games (incl multiplayer games), social media, certain telehealth applications, and other interactive and/or networked electronic applications. Some of the items (e.g. all subitems under item 5 - description of the intervention) may also be applicable for other study designs.

The goal of the CONSORT EHEALTH checklist and guideline is to be
a) a guide for reporting for authors of RCTs,
b) to form a basis for appraisal of an ehealth trial (in terms of validity)

CONSORT-EHEALTH items/subitems are MANDATORY reporting items for studies published in the Journal of Medical Internet Research and other journals / scientific societies endorsing the checklist.

Items numbered 1., 2., 3., 4a., 4b etc are original CONSORT or CONSORT-NPT (non-pharmacologic treatment) items.
Items with Roman numerals (i., ii, iii, iv etc.) are CONSORT-EHEALTH extensions/clarifications.

As the CONSORT-EHEALTH checklist is still considered in a formative stage, we would ask that you also RATE ON A SCALE OF 1-5 how important/useful you feel each item is FOR THE PURPOSE OF THE CHECKLIST and reporting guideline (optional).

Mandatory reporting items are marked with a red *.
In the textboxes, either copy & paste the relevant sections from your manuscript into this form - please include any quotes from your manuscript in QUOTATION MARKS,
or answer directly by providing additional information not in the manuscript, or elaborating on why the item was not relevant for this study.

YOUR ANSWERS WILL BE PUBLISHED AS A SUPPLEMENTARY FILE TO YOUR PUBLICATION IN JMIR AND ARE CONSIDERED PART OF YOUR PUBLICATION (IF ACCEPTED).
Please fill in these questions diligently. Information will not be copyedited, so please use proper spelling and grammar, use correct capitalization, and avoid abbreviations.

DO NOT FORGET TO SAVE AS PDF _AND_ CLICK THE SUBMIT BUTTON SO YOUR ANSWERS ARE IN OUR DATABASE !!!

Citation Suggestion (if you append the pdf as Appendix we suggest to cite this paper in the caption):
Eysenbach G, CONSORT-EHEALTH Group
CONSORT-EHEALTH: Improving and Standardizing Evaluation Reports of Web-based and Mobile Health Interventions
J Med Internet Res 2011;13(4):e126
URL: http://www.jmir.org/2011/4/e126/
doi: 10.2196/jmir.1923
PMID: 22209829

riko.uwagawa@gmail.com
アカウントを切り替える
下書きを保存しました
共有なし

* 必須の質問です

Your name *
First Last

Riko Uwagawa

Primary Affiliation (short), City, Country *
University of Toronto, Toronto, Canada

The University of Tokyo, Tokyo, Japan

Your e-mail address *
abc@gmail.com

ruwagawa1125@gmail.com

Title of your manuscript *

Provide the (draft) title of your manuscript.

Effects of an 8-Week App-Based Mindfulness Intervention on Mental Health in Working Women: Randomized Controlled Trial

Name of your App/Software/Intervention *

If there is a short and a long/alternate name, write the short name first and add the long name in brackets.

No specific name was assigned.

Evaluated Version (if any)

e.g. "V1", "Release 2017-03-01", "Version 2.0.27913"

回答を入力

Language(s) *

What language is the intervention/app in? If multiple languages are available, separate by comma (e.g. "English, French")

Japanese

URL of your Intervention Website or App

e.g. a direct link to the mobile app on app in appstore (itunes, Google Play), or URL of the website. If the intervention is a DVD or hardware, you can also link to an Amazon page.

回答を入力

URL of an image/screenshot (optional)

回答を入力

Accessibility *

Can an enduser access the intervention presently?

- ○ access is free and open
- ◉ access only for special usergroups, not open
- ○ access is open to everyone, but requires payment/subscription/in-app purchases
- ○ app/intervention no longer accessible
- ○ その他:

Primary Medical Indication/Disease/Condition *

e.g. "Stress", "Diabetes", or define the target group in brackets after the condition, e.g. "Autism (Parents of children with)", "Alzheimers (Informal Caregivers of)"

Stress

Primary Outcomes measured in trial *

comma-separated list of primary outcomes reported in the trial

Life satisfaction, Perceived stress, Depressive

Secondary/other outcomes

Are there any other outcomes the intervention is expected to affect?

回答を入力

Recommended "Dose" *

What do the instructions for users say on how often the app should be used?

- (selected) Approximately Daily
- Approximately Weekly
- Approximately Monthly
- Approximately Yearly
- "as needed"
- その他:

Approx. Percentage of Users (starters) still using the app as recommended after 3 months *

- (●) unknown / not evaluated
- ( ) 0-10%
- ( ) 11-20%
- ( ) 21-30%
- ( ) 31-40%
- ( ) 41-50%
- ( ) 51-60%
- ( ) 61-70%
- ( ) 71%-80%
- ( ) 81-90%
- ( ) 91-100%
- ( ) その他:

Overall, was the app/intervention effective? *

- ( ) yes: all primary outcomes were significantly better in intervention group vs control
- (•) partly: SOME primary outcomes were significantly better in intervention group vs control
- ( ) no statistically significant difference between control and intervention
- ( ) potentially harmful: control was significantly better than intervention in one or more outcomes
- ( ) inconclusive: more research is needed
- ( ) その他:

Article Preparation Status/Stage *

At which stage in your article preparation are you currently (at the time you fill in this form)

- ( ) not submitted yet - in early draft status
- ( ) not submitted yet - in late draft status, just before submission
- ( ) submitted to a journal but not reviewed yet
- ( ) submitted to a journal and after receiving initial reviewer comments
- (•) submitted to a journal and accepted, but not published yet
- ( ) published
- ( ) その他:

Journal *

If you already know where you will submit this paper (or if it is already submitted), please provide the journal name (if it is not JMIR, provide the journal name under "other")

- [ ] not submitted yet / unclear where I will submit this
- [x] Journal of Medical Internet Research (JMIR)
- [ ] JMIR mHealth and UHealth
- [ ] JMIR Serious Games
- [ ] JMIR Mental Health
- [ ] JMIR Public Health
- [ ] JMIR Formative Research
- [ ] Other JMIR sister journal
- [ ] その他:

Is this a full powered effectiveness trial or a pilot/feasibility trial? *

- [x] Pilot/feasibility
- [ ] Fully powered

Manuscript tracking number *

If this is a JMIR submission, please provide the manuscript tracking number under "other" (The ms tracking number can be found in the submission acknowledgement email, or when you login as author in JMIR. If the paper is already published in JMIR, then the ms tracking number is the four-digit number at the end of the DOI, to be found at the bottom of each published article in JMIR)

- ○ no ms number (yet) / not (yet) submitted to / published in JMIR
- ◉ その他: 62814

TITLE AND ABSTRACT

1a) TITLE: Identification as a randomized trial in the title

1a) Does your paper address CONSORT item 1a? *

I.e does the title contain the phrase "Randomized Controlled Trial"? (if not, explain the reason under "other")

- ◉ yes
- ○ その他:

1a-i) Identify the mode of delivery in the title

Identify the mode of delivery. Preferably use “web-based” and/or “mobile” and/or “electronic game” in the title. Avoid ambiguous terms like “online”, “virtual”, “interactive”. Use “Internet-based” only if Intervention includes non-web-based Internet components (e.g. email), use “computer-based” or “electronic” only if offline products are used. Use “virtual” only in the context of “virtual reality” (3-D worlds). Use “online” only in the context of “online support groups”. Complement or substitute product names with broader terms for the class of products (such as “mobile” or “smart phone” instead of “iphone”), especially if the application runs on different platforms.

	1	2	3	4	5	
subitem not at all important	○	○	○	○	○	essential

Does your paper address subitem 1a-i? *

Copy and paste relevant sections from manuscript title (include quotes in quotation marks "like this" to indicate direct quotes from your manuscript), or elaborate on this item by providing additional information not in the ms, or briefly explain why the item is not applicable/relevant for your study

App-Based Mindfulness Intervention

1a-ii) Non-web-based components or important co-interventions in title

Mention non-web-based components or important co-interventions in title, if any (e.g., “with telephone support”).

	1	2	3	4	5	
subitem not at all important	○	○	○	○	○	essential

Does your paper address subitem 1a-ii?

Copy and paste relevant sections from manuscript title (include quotes in quotation marks "like this" to indicate direct quotes from your manuscript), or elaborate on this item by providing additional information not in the ms, or briefly explain why the item is not applicable/relevant for your study

回答を入力

1a-iii) Primary condition or target group in the title

Mention primary condition or target group in the title, if any (e.g., “for children with Type I Diabetes”) Example: A Web-based and Mobile Intervention with Telephone Support for Children with Type I Diabetes: Randomized Controlled Trial

	1	2	3	4	5	
subitem not at all important	○	○	○	○	○	essential

Does your paper address subitem 1a-iii? *

Copy and paste relevant sections from manuscript title (include quotes in quotation marks "like this" to indicate direct quotes from your manuscript), or elaborate on this item by providing additional information not in the ms, or briefly explain why the item is not applicable/relevant for your study

Effects of an 8-Week App-Based Mindfulness Intervention on Mental Health in Working Women: Randomized Controlled Trial

1b) ABSTRACT: Structured summary of trial design, methods, results, and conclusions

NPT extension: Description of experimental treatment, comparator, care providers, centers, and blinding status.

1b-i) Key features/functionalities/components of the intervention and comparator in the METHODS section of the ABSTRACT

Mention key features/functionalities/components of the intervention and comparator in the abstract. If possible, also mention theories and principles used for designing the site. Keep in mind the needs of systematic reviewers and indexers by including important synonyms. (Note: Only report in the abstract what the main paper is reporting. If this information is missing from the main body of text, consider adding it)

	1	2	3	4	5	
subitem not at all important	○	○	○	○	○	essential

Does your paper address subitem 1b-i? *

Copy and paste relevant sections from the manuscript abstract (include quotes in quotation marks "like this" to indicate direct quotes from your manuscript), or elaborate on this item by providing additional information not in the ms, or briefly explain why the item is not applicable/relevant for your study

This study recruited women workers via various media sources, such as crowdsourcing sites and social networking services.

1b-ii) Level of human involvement in the METHODS section of the ABSTRACT

Clarify the level of human involvement in the abstract, e.g., use phrases like “fully automated” vs. “therapist/nurse/care provider/physician-assisted” (mention number and expertise of providers involved, if any). (Note: Only report in the abstract what the main paper is reporting. If this information is missing from the main body of text, consider adding it)

	1	2	3	4	5	
subitem not at all important	○	○	○	○	○	essential

Does your paper address subitem 1b-ii?

Copy and paste relevant sections from the manuscript abstract (include quotes in quotation marks "like this" to indicate direct quotes from your manuscript), or elaborate on this item by providing additional information not in the ms, or briefly explain why the item is not applicable/relevant for your study

回答を入力

1b-iii) Open vs. closed, web-based (self-assessment) vs. face-to-face assessments in the METHODS section of the ABSTRACT

Mention how participants were recruited (online vs. offline), e.g., from an open access website or from a clinic or a closed online user group (closed usergroup trial), and clarify if this was a purely web-based trial, or there were face-to-face components (as part of the intervention or for assessment). Clearly say if outcomes were self-assessed through questionnaires (as common in web-based trials). Note: In traditional offline trials, an open trial (open-label trial) is a type of clinical trial in which both the researchers and participants know which treatment is being administered. To avoid confusion, use “blinded” or “unblinded” to indicated the level of blinding instead of “open”, as “open” in web-based trials usually refers to “open access” (i.e. participants can self-enrol). (Note: Only report in the abstract what the main paper is reporting. If this information is missing from the main body of text, consider adding it)

	1	2	3	4	5	
subitem not at all important	○	○	○	○	○	essential

Does your paper address subitem 1b-iii?

Copy and paste relevant sections from the manuscript abstract (include quotes in quotation marks "like this" to indicate direct quotes from your manuscript), or elaborate on this item by providing additional information not in the ms, or briefly explain why the item is not applicable/relevant for your study

回答を入力

1b-iv) RESULTS section in abstract must contain use data

Report number of participants enrolled/assessed in each group, the use/uptake of the intervention (e.g., attrition/adherence metrics, use over time, number of logins etc.), in addition to primary/secondary outcomes. (Note: Only report in the abstract what the main paper is reporting. If this information is missing from the main body of text, consider adding it)

	1	2	3	4	5	
subitem not at all important	○	○	○	○	○	essential

Does your paper address subitem 1b-iv?

Copy and paste relevant sections from the manuscript abstract (include quotes in quotation marks "like this" to indicate direct quotes from your manuscript), or elaborate on this item by providing additional information not in the ms, or briefly explain why the item is not applicable/relevant for your study

回答を入力

1b-v) CONCLUSIONS/DISCUSSION in abstract for negative trials

Conclusions/Discussions in abstract for negative trials: Discuss the primary outcome - if the trial is negative (primary outcome not changed), and the intervention was not used, discuss whether negative results are attributable to lack of uptake and discuss reasons. (Note: Only report in the abstract what the main paper is reporting. If this information is missing from the main body of text, consider adding it)

	1	2	3	4	5	
subitem not at all important	○	○	○	○	○	essential

Does your paper address subitem 1b-v?

Copy and paste relevant sections from the manuscript abstract (include quotes in quotation marks "like this" to indicate direct quotes from your manuscript), or elaborate on this item by providing additional information not in the ms, or briefly explain why the item is not applicable/relevant for your study

回答を入力

INTRODUCTION

2a) In INTRODUCTION: Scientific background and explanation of rationale

2a-i) Problem and the type of system/solution

Describe the problem and the type of system/solution that is object of the study: intended as stand-alone intervention vs. incorporated in broader health care program? Intended for a particular patient population? Goals of the intervention, e.g., being more cost-effective to other interventions, replace or complement other solutions? (Note: Details about the intervention are provided in “Methods” under 5)

	1	2	3	4	5	
subitem not at all important	○	○	○	○	○	essential

Does your paper address subitem 2a-i? *

Copy and paste relevant sections from the manuscript (include quotes in quotation marks "like this" to indicate direct quotes from your manuscript), or elaborate on this item by providing additional information not in the ms, or briefly explain why the item is not applicable/relevant for your study

This is a stand-alone intervention using four meditation content programs. While the intervention itself is not targeted at a particular patient population, the target population of this study is working women.

2a-ii) Scientific background, rationale: What is known about the (type of) system

Scientific background, rationale: What is known about the (type of) system that is the object of the study (be sure to discuss the use of similar systems for other conditions/diagnoses, if appropiate), motivation for the study, i.e. what are the reasons for and what is the context for this specific study, from which stakeholder viewpoint is the study performed, potential impact of findings [2]. Briefly justify the choice of the comparator.

	1	2	3	4	5	
subitem not at all important	○	○	○	○	○	essential

Does your paper address subitem 2a-ii? *

Copy and paste relevant sections from the manuscript (include quotes in quotation marks "like this" to indicate direct quotes from your manuscript), or elaborate on this item by providing additional information not in the ms, or briefly explain why the item is not applicable/relevant for your study

回答を入力

2b) In INTRODUCTION: Specific objectives or hypotheses

Does your paper address CONSORT subitem 2b? *

Copy and paste relevant sections from the manuscript (include quotes in quotation marks "like this" to indicate direct quotes from your manuscript), or elaborate on this item by providing additional information not in the ms, or briefly explain why the item is not applicable/relevant for your study

"Therefore, this study aimed to evaluate the effectiveness of an 8-week mindfulness meditation intervention via a smartphone app among women workers through a randomized controlled trial (RCT). Effectiveness was examined via 4 indicators: general psychological, work-related, family-related, and work-to-conflict measures. Furthermore, we examined the measures that would effectively influence. We hypothesized that participants in the intervention group (self-care mindfulness meditation via the smartphone app) would have a higher level of general psychological (life satisfaction, perceived stress, depressive and anxiety symptoms, trait anger, and mindfulness), work-related (work performance, job satisfaction, quantitative job overload, and job control), family-related (family satisfaction and partner satisfaction), and work-to-family conflict indicators compared with those in the waitlist control group."

METHODS

3a) Description of trial design (such as parallel, factorial) including allocation ratio

Does your paper address CONSORT subitem 3a? *

Copy and paste relevant sections from the manuscript (include quotes in quotation marks "like this" to indicate direct quotes from your manuscript), or elaborate on this item by providing additional information not in the ms, or briefly explain why the item is not applicable/relevant for your study

"Procedure
Overview
This study was designed as a parallel-design RCT. Randomization was computerized independently by research staff using a blocked randomization scheme (block size 10). Participants were expected to be randomized in a ratio of 1:1 to the intervention or waitlist control group. This study was an open-label RCT as it was not possible to blind the allocation."

3b) Important changes to methods after trial commencement (such as eligibility criteria), with reasons

Does your paper address CONSORT subitem 3b? *

Copy and paste relevant sections from the manuscript (include quotes in quotation marks "like this" to indicate direct quotes from your manuscript), or elaborate on this item by providing additional information not in the ms, or briefly explain why the item is not applicable/relevant for your study

We do not change methods after trial commencement.

3b-i) Bug fixes, Downtimes, Content Changes

Bug fixes, Downtimes, Content Changes: ehealth systems are often dynamic systems. A description of changes to methods therefore also includes important changes made on the intervention or comparator during the trial (e.g., major bug fixes or changes in the functionality or content) (5-iii) and other “unexpected events” that may have influenced study design such as staff changes, system failures/downtimes, etc. [2].

	1	2	3	4	5	
subitem not at all important	○	○	○	○	○	essential

Does your paper address subitem 3b-i?

Copy and paste relevant sections from the manuscript (include quotes in quotation marks "like this" to indicate direct quotes from your manuscript), or elaborate on this item by providing additional information not in the ms, or briefly explain why the item is not applicable/relevant for your study

回答を入力

4a) Eligibility criteria for participants

Does your paper address CONSORT subitem 4a? *

Copy and paste relevant sections from the manuscript (include quotes in quotation marks "like this" to indicate direct quotes from your manuscript), or elaborate on this item by providing additional information not in the ms, or briefly explain why the item is not applicable/relevant for your study

This study recruited 397 women workers via various media sources, such as crowdsourcing sites and social networking services. Inclusion criteria included those who were (1) biologically female, (2) employed for at least 20 hours per week,(3) owned an iPhone (for convenience of the app used), and (4) aged 18-64 years. Exclusion criteria included those who (1) received treatment for a mental disorder, (2) scored ≥13 on the 6-item Kessler Psychological Distress Scale (K6) Japanese version, (3) were on leave, and (4) were currently pregnant or likely to become pregnant within six months. Among the participants, 95 did not meet the inclusion and exclusion criteria. Hence, 302 women workers who met the criteria were asked to respond to the preintervention assessment, and 215 who completed the assessment were randomly assigned to the intervention (n=107) or waitlist control group (n=108). Randomization was computerized using a blocked randomization scheme (block size 10). A total of 8 working women dropped out. Of the 8 participants, 2 participants (intervention group, n=1; wait-list control group, n=1) declined to participate in this study, 2 participants in the intervention group opted out of the intervention, and 4 participants (intervention group, n=2; wait-list control group, n=2) could not be contacted. After 8 weeks, the participants were asked to respond to the postintervention assessment, and 196 women workers completed the assessment (intervention group, n=95; waitlist control group, n=101). Of 215 participants who completed the preintervention assessment, 4 who worked <19 hours per week on average in the preintervention assessment were excluded from analysis (intervention group, n=1; waitlist control group, n=3). Therefore, of the 215 participants who were randomized, data from 209 participants (intervention group, n=105; waitlist control group, n=104) were finally analyzed, excluding 2 participants who declined to participate in this study and 4 participants who worked <19 hours per week on average in the preintervention assessment.

4a-i) Computer / Internet literacy

Computer / Internet literacy is often an implicit “de facto” eligibility criterion - this should be explicitly clarified.

	1	2	3	4	5	
subitem not at all important	○	○	○	○	○	essential

Does your paper address subitem 4a-i?

Copy and paste relevant sections from the manuscript (include quotes in quotation marks "like this" to indicate direct quotes from your manuscript), or elaborate on this item by providing additional information not in the ms, or briefly explain why the item is not applicable/relevant for your study

回答を入力

4a-ii) Open vs. closed, web-based vs. face-to-face assessments:

Open vs. closed, web-based vs. face-to-face assessments: Mention how participants were recruited (online vs. offline), e.g., from an open access website or from a clinic, and clarify if this was a purely web-based trial, or there were face-to-face components (as part of the intervention or for assessment), i.e., to what degree got the study team to know the participant. In online-only trials, clarify if participants were quasi-anonymous and whether having multiple identities was possible or whether technical or logistical measures (e.g., cookies, email confirmation, phone calls) were used to detect/prevent these.

	1	2	3	4	5	
subitem not at all important	○	○	○	○	○	essential

Does your paper address subitem 4a-ii? *

Copy and paste relevant sections from the manuscript (include quotes in quotation marks "like this" to indicate direct quotes from your manuscript), or elaborate on this item by providing additional information not in the ms, or briefly explain why the item is not applicable/relevant for your study

This study recruited 397 women workers via various media sources, such as crowdsourcing sites and social networking services. Inclusion criteria included those who were (1) biologically female, (2) employed for at least 20 hours per week,(3) owned an iPhone (for convenience of the app used), and (4) aged 18-64 years. Exclusion criteria included those who (1) received treatment for a mental disorder, (2) scored ≥13 on the 6-item Kessler Psychological Distress Scale (K6) Japanese version, (3) were on leave, and (4) were currently pregnant or likely to become pregnant within six months. Among the participants, 95 did not meet the inclusion and exclusion criteria. Hence, 302 women workers who met the criteria were asked to respond to the preintervention assessment, and 215 who completed the assessment were randomly assigned to the intervention (n=107) or waitlist control group (n=108). Randomization was computerized using a blocked randomization scheme (block size 10). A total of 8 working women dropped out. Of the 8 participants, 2 participants (intervention group, n=1; wait-list control group, n=1) declined to participate in this study, 2 participants in the intervention group opted out of the intervention, and 4 participants (intervention group, n=2; wait-list control group, n=2) could not be contacted. After 8 weeks, the participants were asked to respond to the postintervention assessment, and 196 women workers completed the assessment (intervention group, n=95; waitlist control group, n=101). Of 215 participants who completed the preintervention assessment, 4 who worked <19 hours per week on average in the preintervention assessment were excluded from analysis (intervention group, n=1; waitlist control group, n=3). Therefore, of the 215 participants who were randomized, data from 209 participants (intervention group, n=105; waitlist control group, n=104) were finally analyzed, excluding 2 participants who declined to participate in this study and 4 participants who worked <19 hours per week on average in the preintervention assessment.

4a-iii) Information giving during recruitment

Information given during recruitment. Specify how participants were briefed for recruitment and in the informed consent procedures (e.g., publish the informed consent documentation as appendix, see also item X26), as this information may have an effect on user self-selection, user expectation and may also bias results.

	1	2	3	4	5	
subitem not at all important	○	○	○	○	○	essential

Does your paper address subitem 4a-iii?

Copy and paste relevant sections from the manuscript (include quotes in quotation marks "like this" to indicate direct quotes from your manuscript), or elaborate on this item by providing additional information not in the ms, or briefly explain why the item is not applicable/relevant for your study

回答を入力

4b) Settings and locations where the data were collected

Does your paper address CONSORT subitem 4b? *

Copy and paste relevant sections from the manuscript (include quotes in quotation marks "like this" to indicate direct quotes from your manuscript), or elaborate on this item by providing additional information not in the ms, or briefly explain why the item is not applicable/relevant for your study

Data were collected online using Google Forms.

4b-i) Report if outcomes were (self-)assessed through online questionnaires

Clearly report if outcomes were (self-)assessed through online questionnaires (as common in web-based trials) or otherwise.

	1	2	3	4	5	
subitem not at all important	○	○	○	○	○	essential

Does your paper address subitem 4b-i? *

Copy and paste relevant sections from the manuscript (include quotes in quotation marks "like this" to indicate direct quotes from your manuscript), or elaborate on this item by providing additional information not in the ms, or briefly explain why the item is not applicable/relevant for your study

This study recruited 397 women workers via various media sources, such as crowdsourcing sites and social networking services. Inclusion criteria included those who were (1) biologically female, (2) employed for at least 20 hours per week,(3) owned an iPhone (for convenience of the app used), and (4) aged 18-64 years. Exclusion criteria included those who (1) received treatment for a mental disorder, (2) scored ≥13 on the 6-item Kessler Psychological Distress Scale (K6) Japanese version, (3) were on leave, and (4) were currently pregnant or likely to become pregnant within six months. Among the participants, 95 did not meet the inclusion and exclusion criteria. Hence, 302 women workers who met the criteria were asked to respond to the preintervention assessment, and 215 who completed the assessment were randomly assigned to the intervention (n=107) or waitlist control group (n=108). Randomization was computerized using a blocked randomization scheme (block size 10). A total of 8 working women dropped out. Of the 8 participants, 2 participants (intervention group, n=1; wait-list control group, n=1) declined to participate in this study, 2 participants in the intervention group opted out of the intervention, and 4 participants (intervention group, n=2; wait-list control group, n=2) could not be contacted. After 8 weeks, the participants were asked to respond to the postintervention assessment, and 196 women workers completed the assessment (intervention group, n=95; waitlist control group, n=101). Of 215 participants who completed the preintervention assessment, 4 who worked <19 hours per week on average in the preintervention assessment were excluded from analysis (intervention group, n=1; waitlist control group, n=3). Therefore, of the 215 participants who were randomized, data from 209 participants (intervention group, n=105; waitlist control group, n=104) were finally analyzed, excluding 2 participants who declined to participate in this study and 4 participants who worked <19 hours per week on average in the preintervention assessment.

4b-ii) Report how institutional affiliations are displayed

Report how institutional affiliations are displayed to potential participants [on ehealth media], as affiliations with prestigious hospitals or universities may affect volunteer rates, use, and reactions with regards to an intervention.(Not a required item – describe only if this may bias results)

	1	2	3	4	5	
subitem not at all important	○	○	○	○	○	essential

Does your paper address subitem 4b-ii?

Copy and paste relevant sections from the manuscript (include quotes in quotation marks "like this" to indicate direct quotes from your manuscript), or elaborate on this item by providing additional information not in the ms, or briefly explain why the item is not applicable/relevant for your study

回答を入力

5) The interventions for each group with sufficient details to allow replication, including how and when they were actually administered

5-i) Mention names, credential, affiliations of the developers, sponsors, and owners

Mention names, credential, affiliations of the developers, sponsors, and owners [6] (if authors/evaluators are owners or developer of the software, this needs to be declared in a “Conflict of interest” section or mentioned elsewhere in the manuscript).

	1	2	3	4	5	
subitem not at all important	○	○	○	○	○	essential

Does your paper address subitem 5-i?

Copy and paste relevant sections from the manuscript (include quotes in quotation marks "like this" to indicate direct quotes from your manuscript), or elaborate on this item by providing additional information not in the ms, or briefly explain why the item is not applicable/relevant for your study

回答を入力

5-ii) Describe the history/development process

Describe the history/development process of the application and previous formative evaluations (e.g., focus groups, usability testing), as these will have an impact on adoption/use rates and help with interpreting results.

	1	2	3	4	5	
subitem not at all important	○	○	○	○	○	essential

Does your paper address subitem 5-ii?

Copy and paste relevant sections from the manuscript (include quotes in quotation marks "like this" to indicate direct quotes from your manuscript), or elaborate on this item by providing additional information not in the ms, or briefly explain why the item is not applicable/relevant for your study

回答を入力

5-iii) Revisions and updating

Revisions and updating. Clearly mention the date and/or version number of the application/intervention (and comparator, if applicable) evaluated, or describe whether the intervention underwent major changes during the evaluation process, or whether the development and/or content was “frozen” during the trial. Describe dynamic components such as news feeds or changing content which may have an impact on the replicability of the intervention (for unexpected events see item 3b).

	1	2	3	4	5	
subitem not at all important	○	○	○	○	○	essential

Does your paper address subitem 5-iii?

Copy and paste relevant sections from the manuscript (include quotes in quotation marks "like this" to indicate direct quotes from your manuscript), or elaborate on this item by providing additional information not in the ms, or briefly explain why the item is not applicable/relevant for your study

回答を入力

5-iv) Quality assurance methods

Provide information on quality assurance methods to ensure accuracy and quality of information provided [1], if applicable.

	1	2	3	4	5	
subitem not at all important	○	○	○	○	○	essential

Does your paper address subitem 5-iv?

Copy and paste relevant sections from the manuscript (include quotes in quotation marks "like this" to indicate direct quotes from your manuscript), or elaborate on this item by providing additional information not in the ms, or briefly explain why the item is not applicable/relevant for your study

回答を入力

5-v) Ensure replicability by publishing the source code, and/or providing screenshots/screen-capture video, and/or providing flowcharts of the algorithms used

Ensure replicability by publishing the source code, and/or providing screenshots/screen-capture video, and/or providing flowcharts of the algorithms used. Replicability (i.e., other researchers should in principle be able to replicate the study) is a hallmark of scientific reporting.

	1	2	3	4	5	
subitem not at all important	○	○	○	○	○	essential

Does your paper address subitem 5-v?

Copy and paste relevant sections from the manuscript (include quotes in quotation marks "like this" to indicate direct quotes from your manuscript), or elaborate on this item by providing additional information not in the ms, or briefly explain why the item is not applicable/relevant for your study

回答を入力

5-vi) Digital preservation

Digital preservation: Provide the URL of the application, but as the intervention is likely to change or disappear over the course of the years; also make sure the intervention is archived (Internet Archive, webcitation.org, and/or publishing the source code or screenshots/videos alongside the article). As pages behind login screens cannot be archived, consider creating demo pages which are accessible without login.

	1	2	3	4	5	
subitem not at all important	○	○	○	○	○	essential

Does your paper address subitem 5-vi?

Copy and paste relevant sections from the manuscript (include quotes in quotation marks "like this" to indicate direct quotes from your manuscript), or elaborate on this item by providing additional information not in the ms, or briefly explain why the item is not applicable/relevant for your study

回答を入力

5-vii) Access

Access: Describe how participants accessed the application, in what setting/context, if they had to pay (or were paid) or not, whether they had to be a member of specific group. If known, describe how participants obtained “access to the platform and Internet” [1]. To ensure access for editors/reviewers/readers, consider to provide a “backdoor” login account or demo mode for reviewers/readers to explore the application (also important for archiving purposes, see vi).

	1	2	3	4	5	
subitem not at all important	○	○	○	○	○	essential

Does your paper address subitem 5-vii? *

Copy and paste relevant sections from the manuscript (include quotes in quotation marks "like this" to indicate direct quotes from your manuscript), or elaborate on this item by providing additional information not in the ms, or briefly explain why the item is not applicable/relevant for your study

回答を入力

5-viii) Mode of delivery, features/functionalities/components of the intervention and comparator, and the theoretical framework

Describe mode of delivery, features/functionalities/components of the intervention and comparator, and the theoretical framework [6] used to design them (instructional strategy [1], behaviour change techniques, persuasive features, etc., see e.g., [7, 8] for terminology). This includes an in-depth description of the content (including where it is coming from and who developed it) [1],” whether [and how] it is tailored to individual circumstances and allows users to track their progress and receive feedback” [6]. This also includes a description of communication delivery channels and – if computer-mediated communication is a component – whether communication was synchronous or asynchronous [6]. It also includes information on presentation strategies [1], including page design principles, average amount of text on pages, presence of hyperlinks to other resources, etc. [1].

	1	2	3	4	5	
subitem not at all important	○	○	○	○	○	essential

Does your paper address subitem 5-viii? *

Copy and paste relevant sections from the manuscript (include quotes in quotation marks "like this" to indicate direct quotes from your manuscript), or elaborate on this item by providing additional information not in the ms, or briefly explain why the item is not applicable/relevant for your study

"8-Week Mindfulness-Based Self-Help Intervention via the Smartphone App
Mindfulness meditation was conducted via the iOS app, with the content changed every 2 weeks (Table 1). The app displayed the day's meditation content and explanation on the home screen. After viewing this screen, the participants pressed the play button to hear the guided audio and practiced meditation. Figure 1 illustrates the display of the app. In addition, the psychoeducation pages on mindfulness and self-compassion were created and inserted on the app (Figure 2).
The content included "meditation of breath," "meditation of breath, sound, and body," and "body scan meditation," based on previous studies [22]. As the "body scan" was partially included in "meditation of breath, sound, and body," in this study, the latter was conducted after the former. Furthermore, as the effectiveness of interventions that incorporated elements of self-compassion was recently highlighted, "loving-kindness meditation" was ultimately added. As a daily 13-minute meditation was effective after 8 weeks [23], the intervention period was designed to be 8 weeks."

"Results
Baseline
Figure 3 illustrates the CONSORT (Consolidated Standards for Reporting Trials) flow diagram (the CONSORT checklist is provided in Multimedia Appendix 1).
Table 2 shows the participants' demographic characteristics. Chi-squared and t tests revealed no differences in demographic variables and psychological indices between the intervention and waitlist control groups (P>.05)."

5-ix) Describe use parameters

Describe use parameters (e.g., intended "doses" and optimal timing for use). Clarify what instructions or recommendations were given to the user, e.g., regarding timing, frequency, heaviness of use, if any, or was the intervention used ad libitum.

	1	2	3	4	5	
subitem not at all important	○	○	○	○	○	essential

Does your paper address subitem 5-ix?

Copy and paste relevant sections from the manuscript (include quotes in quotation marks "like this" to indicate direct quotes from your manuscript), or elaborate on this item by providing additional information not in the ms, or briefly explain why the item is not applicable/relevant for your study

回答を入力

5-x) Clarify the level of human involvement

Clarify the level of human involvement (care providers or health professionals, also technical assistance) in the e-intervention or as co-intervention (detail number and expertise of professionals involved, if any, as well as “type of assistance offered, the timing and frequency of the support, how it is initiated, and the medium by which the assistance is delivered”. It may be necessary to distinguish between the level of human involvement required for the trial, and the level of human involvement required for a routine application outside of a RCT setting (discuss under item 21 – generalizability).

	1	2	3	4	5	
subitem not at all important	◯	◯	◯	◯	◯	essential

Does your paper address subitem 5-x?

Copy and paste relevant sections from the manuscript (include quotes in quotation marks "like this" to indicate direct quotes from your manuscript), or elaborate on this item by providing additional information not in the ms, or briefly explain why the item is not applicable/relevant for your study

回答を入力

5-xi) Report any prompts/reminders used

Report any prompts/reminders used: Clarify if there were prompts (letters, emails, phone calls, SMS) to use the application, what triggered them, frequency etc. It may be necessary to distinguish between the level of prompts/reminders required for the trial, and the level of prompts/reminders for a routine application outside of a RCT setting (discuss under item 21 – generalizability).

	1	2	3	4	5	
subitem not at all important	○	○	○	○	○	essential

Does your paper address subitem 5-xi? *

Copy and paste relevant sections from the manuscript (include quotes in quotation marks "like this" to indicate direct quotes from your manuscript), or elaborate on this item by providing additional information not in the ms, or briefly explain why the item is not applicable/relevant for your study

If participants had set reminders, they would receive a reminder from the app at the specified time each day.

5-xii) Describe any co-interventions (incl. training/support)

Describe any co-interventions (incl. training/support): Clearly state any interventions that are provided in addition to the targeted eHealth intervention, as ehealth intervention may not be designed as stand-alone intervention. This includes training sessions and support [1]. It may be necessary to distinguish between the level of training required for the trial, and the level of training for a routine application outside of a RCT setting (discuss under item 21 – generalizability.

	1	2	3	4	5	
subitem not at all important	○	○	○	○	○	essential

Does your paper address subitem 5-xii? *

Copy and paste relevant sections from the manuscript (include quotes in quotation marks "like this" to indicate direct quotes from your manuscript), or elaborate on this item by providing additional information not in the ms, or briefly explain why the item is not applicable/relevant for your study

"8-Week Mindfulness-Based Self-Help Intervention via the Smartphone App
Mindfulness meditation was conducted via the iOS app, with the content changed every 2 weeks (Table 1). The app displayed the day's meditation content and explanation on the home screen. After viewing this screen, the participants pressed the play button to hear the guided audio and practiced meditation. Figure 1 illustrates the display of the app. In addition, the psychoeducation pages on mindfulness and self-compassion were created and inserted on the app (Figure 2).
The content included "meditation of breath," "meditation of breath, sound, and body," and "body scan meditation," based on previous studies [22]. As the "body scan" was partially included in "meditation of breath, sound, and body," in this study, the latter was conducted after the former. Furthermore, as the effectiveness of interventions that incorporated elements of self-compassion was recently highlighted, "loving-kindness meditation" was ultimately added. As a daily 13-minute meditation was effective after 8 weeks [23], the intervention period was designed to be 8 weeks."

6a) Completely defined pre-specified primary and secondary outcome measures, including how and when they were assessed

Does your paper address CONSORT subitem 6a? *

Copy and paste relevant sections from the manuscript (include quotes in quotation marks "like this" to indicate direct quotes from your manuscript), or elaborate on this item by providing additional information not in the ms, or briefly explain why the item is not applicable/relevant for your study

"Procedure
Overview
This study was designed as a parallel-design RCT. Randomization was computerized independently by research staff using a blocked randomization scheme (block size 10). Participants were expected to be randomized in a ratio of 1:1 to the intervention or waitlist control group. This study was an open-label RCT as it was not possible to blind the allocation.
This study was conducted from July 2023 to January 2024 via web forms. Participants in

the intervention group installed the app for meditation after the preintervention assessment. Participants practiced guided mindfulness meditation via the app on their cell phones every day at their convenience for 8 weeks. After 8 weeks, the participants received the postintervention questionnaire via the app and email. The participants in the waitlist control group lived as usual for 8 weeks after the preintervention assessment. After 8 weeks, they also responded to the postintervention questionnaire via email."

"Measurements
General Psychological Domain: Well-Being
Well-being was assessed as life satisfaction using the 5-item Satisfaction with Life Scale. Participants evaluated their subjective life satisfaction on a 7-point Likert scale that ranged from 1 (strongly disagree) to 7 (strongly agree) [24,25]. This measurement was developed by Diener et al [24]. The development of the Japanese version used in this study and its validity and reliability were studied by Sumino [25]. Sample items included "In most ways, my life is close to my ideal." The total score was a sum of all the individual item scores, and higher scores indicated greater life satisfaction. In this study, Cronbach α was 0.85 and 0.81 for the pre- and postintervention assessments, respectively.
Mental Health Outcomes
Perceived Stress
The 10-item Perceived Stress Scale was used to assess perceived stress. Participants rated how unpredictable, uncontrollable, and overloaded they found their lives on a 5-point Likert scale that ranged from 0 (never) to 4 (very often) [26,27]. This measurement was developed by Cohen et al [26]. The development of the Japanese version used in this study and its validity and reliability were studied by Sumi [27]. Sample items included "How often have you been upset because of something that happened unexpectedly?" The total score was a sum of the individual item scores, and higher scores indicated greater perceived stress. Cronbach α was 0.69 and 0.79 for pre- and postintervention, respectively.
Depressive and Anxiety Symptoms
K6 was used to assess depression and anxiety symptoms. Participants described how often they experienced depressive symptoms in the past 30 days on a 5-point Likert scale that ranged from 0 (none of the time) to 4 (all of the time) [28-30]. This measurement was developed by Kessler et al [28], and the Japanese version of it used in this study was developed by Furukawa et al [29]. The validity and reliability were studied by Furukawa et al [29] and by Sakurai et al [30]. Sample items included "How often did you feel nervous?" and "How often did you feel restless or fidgety?" The total score was a sum of all the individual item scores, and higher scores indicated a greater severity of depression and anxiety. Cronbach α was 0.83 and 0.80 for pre- and postintervention, respectively.
Trait Anger
"Trait anger (T-Ang; 10-item)," a subscale of the 57-item State-Trait Anger Expression Inventory 2 (STAXI-2), was used to assess the traits of anger reaction [31-33]. This measurement was developed by Spielberger [31]. The development of the Japanese version used in this study and its reliability were studied by Mine and Ohki [32] and by Mine and Sato [33]. Participants evaluated their perceptions of anger proneness on a 4-point Likert scale that ranged from 1 (strongly disagree) to 4 (strongly agree). Sample items included "I am quick-tempered." T-Ang included two subfactors: T-Ang/Temperament (T-Ang/T; trait of feeling anger with or without stimulus) and T-Ang/Reaction (T-Ang/R; frequency of

[illegible]
experiencing feelings of anger in situations involving irritation or negative evaluation). The total score within each subfactor and all items was calculated by summing the item scores. Higher scores indicated greater trait anger. Cronbach α for preintervention was T-Ang Cronbach α=0.84, T-Ang/T Cronbach α=0.79, and T-Ang/R Cronbach α=0.77, and for postintervention was T-Ang Cronbach α=0.83, T-Ang/T
Cronbach α=0.84, and T-Ang/R Cronbach α=0.76. Mindfulness
The 15-item Mindful Attention Awareness Scale was used to assess dispositional mindfulness [34,35]. This measurement was developed by Brown and Ryan [34]. The development of the Japanese version used in this study and its validity and reliability were studied by Fujino et al [35]. Participants rated the degree to which they functioned without awareness of the present experience in daily life on a 6-point scale that ranged from 1 (almost never) to 6 (almost always). Sample items included “I could be experiencing some emotion and not be conscious of it until sometime later.” All items were reversed as they assessed the lack of mindful attention and awareness. The total score was a sum of all the reversed-item scores, and higher scores indicated greater mindful attention and awareness. Cronbach α was 0.81 and 0.87 for pre- and postintervention, respectively.
Work-Related Domain
Work Performance
The World Health Organization Health and Work Performance Questionnaire Short Form was used to assess work performance. The questions included: “On a scale of 0-10, where 0 is the worst job performance anyone could have at your job, and 10 is the performance of a top worker, how would you rate the usual performance of most workers in a job similar to yours?” (possible performance) and “Using the same 0-10 scale, how would you rate your overall job performance on the days you worked during the past four weeks?”(actual performance) [36-38]. This measurement was developed by Kessler et al [36]. The development of the Japanese version used in this study and its validity and reliability were studied by Kawakami et al [38]. Participants evaluated the workplace costs of health problems regarding self-reported sickness leaves and reduced job performance (presenteeism). Presenteeism was assessed by “absolute” and “relative presenteeism.” “Absolute presenteeism” was calculated by multiplying the score of actual performance by 10. Higher scores indicated greater performance. “Relative presenteeism” was calculated by the ratio of actual performance to possible performance (restricted to the range of 0.25–2.0, where values <0.25 and >2.0 were converted to 0.25 and 2.0, respectively). Higher scores indicated greater performance.
Job Satisfaction
Job satisfaction was assessed via a single item from the Brief Job Stress Questionnaire (BJSQ) [39]. The development of this measurement used in this study and its validity and reliability were studied by Inoue et al [39]. Participants rated the degree to which they agreed with the item, “I am satisfied with my job,” on a 4-point Likert scale that ranged from 1 (satisfied) to 4 (dissatisfied). The item was reversed as it assessed the high level of job satisfaction. Higher scores indicated greater job satisfaction.
Quantitative Job Overload
“Quantitative job overload (3-item),” a subscale of the BJSQ, was used to assess job overload [39]. Participants rated the degree of their job overload on a 4-point Likert scale that ranged from 1 (agree) to 4 (disagree). Sample items included “I have a lot of work to do.” All items were reversed as they assessed the high level of job overload. The total score

do." All items were reversed as they assessed the high level of job overload. The total score was a sum of all the reversed-item scores, and higher scores indicated a greater job overload. Cronbach α was 0.64 and 0.56 for pre- and postintervention, respectively.
Job Control
"Job control (3-item)," a subscale of the BJSQ, was used to assess job control [39]. Participants rated the degree of their job control on a 4-point Likert scale that ranged from 1 (agree) to 4 (disagree). Sample items included "I can work at my own pace." All items were reversed as they assessed the high level of job control. The total score was a sum of all the reversed-item scores, and higher scores indicated a greater sense of job control. Cronbach α was 0.60 and 0.64 for pre- and postintervention, respectively.
Family-Related Domain
Family Satisfaction
Family satisfaction was assessed via a single item from the BJSQ [39]. Participants rated the degree to which they agreed with the item, "I am satisfied with my family life," on a 4-point Likert scale that ranged from 1 (satisfied) to 4 (dissatisfied). The item was reversed as it assessed the high level of family satisfaction. Higher scores indicated greater family satisfaction.
Partner Satisfaction
Partner satisfaction was assessed via a single item: "Using the 10-point scale, how would you rate your current level of satisfaction with your relationship with your partner?" Only participants who lived with their partners were asked to respond. Participants rated the degree of their satisfaction with their partner on a 10-point Likert scale that ranged from 1 (dissatisfied) to 10 (satisfied). Higher scores indicated greater satisfaction.
Work-to-Family Conflict Domain
The 22-item Survey Work-Home Interaction-Nijmegen was used to assess the 4 subscales that reflected the underlying dimensions of work–family spillover: (1) work-family negative spillover (WFNS, 8 items; eg, "You do not have the energy to engage in leisure activities with your spouse/family/friends because of your job."), (2) family-work negative spillover (FWNS, 4 items; eg, "You do not feel like working because of problems with your spouse/family/friends."), (3) work-family positive spillover (WFPS, 5 items; eg, "You fulfill your domestic obligations better because of the things you have learned on your job."), (4) family-work positive spillover (FWPS, 5 items; eg, "You have greater self-confidence at work because you have your home life well organized") [40,41]. This measurement was developed by Geurts et al [40] in 2005. The development of the Japanese version used in this study and its validity and reliability were studied by Shimada et al [41]. Responses were rated on a 4-point Likert scale that ranged from 0 (never) to 3 (always). The total score of each subscale was calculated as a sum of all the individual item scores. Higher scores on the positive (WFPS and FWPS) and negative spillover subscales (WFNS and FWNS) indicated greater positive and negative impacts, respectively. For preintervention, the Cronbach α were WFNS Cronbach α=0.88, FWNS Cronbach α=0.79, WFPS Cronbach α=0.73, and FWPS Cronbach α=0.78, and for postintervention, it was WFNS Cronbach α=0.89, FWNS Cronbach α=0.76, WFPS Cronbach α=0.79, and FWPS Cronbach α=0.83."

6a-i) Online questionnaires: describe if they were validated for online use and apply CHERRIES items to describe how the questionnaires were designed/deployed

If outcomes were obtained through online questionnaires, describe if they were validated for online use and apply CHERRIES items to describe how the questionnaires were designed/deployed [9].

	1	2	3	4	5	
subitem not at all important	○	○	○	○	○	essential

Does your paper address subitem 6a-i?

Copy and paste relevant sections from manuscript text

回答を入力

6a-ii) Describe whether and how “use” (including intensity of use/dosage) was defined/measured/monitored

Describe whether and how “use” (including intensity of use/dosage) was defined/measured/monitored (logins, logfile analysis, etc.). Use/adoption metrics are important process outcomes that should be reported in any ehealth trial.

	1	2	3	4	5	
subitem not at all important	○	○	○	○	○	essential

Does your paper address subitem 6a-ii?

Copy and paste relevant sections from manuscript text

回答を入力

6a-iii) Describe whether, how, and when qualitative feedback from participants was obtained

Describe whether, how, and when qualitative feedback from participants was obtained (e.g., through emails, feedback forms, interviews, focus groups).

	1	2	3	4	5	
subitem not at all important	○	○	○	○	○	essential

Does your paper address subitem 6a-iii?

Copy and paste relevant sections from manuscript text

回答を入力

6b) Any changes to trial outcomes after the trial commenced, with reasons

Does your paper address CONSORT subitem 6b? *

Copy and paste relevant sections from the manuscript (include quotes in quotation marks "like this" to indicate direct quotes from your manuscript), or elaborate on this item by providing additional information not in the ms, or briefly explain why the item is not applicable/relevant for your study

There was not any changes to trial outcomes after the trial commenced.

7a) How sample size was determined

NPT: When applicable, details of whether and how the clustering by care provides or centers was addressed

7a-i) Describe whether and how expected attrition was taken into account when calculating the sample size

Describe whether and how expected attrition was taken into account when calculating the sample size.

	1	2	3	4	5	
subitem not at all important	○	○	○	○	○	essential

Does your paper address subitem 7a-i?

Copy and paste relevant sections from manuscript title (include quotes in quotation marks "like this" to indicate direct quotes from your manuscript), or elaborate on this item by providing additional information not in the ms, or briefly explain why the item is not applicable/relevant for your study

回答を入力

7b) When applicable, explanation of any interim analyses and stopping guidelines

Does your paper address CONSORT subitem 7b? *

Copy and paste relevant sections from the manuscript (include quotes in quotation marks "like this" to indicate direct quotes from your manuscript), or elaborate on this item by providing additional information not in the ms, or briefly explain why the item is not applicable/relevant for your study

We did not provide an interim analysis or explain the stopping guidelines.

8a) Method used to generate the random allocation sequence

NPT: When applicable, how care providers were allocated to each trial group

Does your paper address CONSORT subitem 8a? *

Copy and paste relevant sections from the manuscript (include quotes in quotation marks "like this" to indicate direct quotes from your manuscript), or elaborate on this item by providing additional information not in the ms, or briefly explain why the item is not applicable/relevant for your study

Procedure
Overview
This study was designed as a parallel-design RCT. Randomization was computerized independently by research staff using a blocked randomization scheme (block size 10). Participants were expected to be randomized in a ratio of 1:1 to the intervention or waitlist control group. This study was an open-label RCT as it was not possible to blind the allocation.

8b) Type of randomisation; details of any restriction (such as blocking and block size)

Does your paper address CONSORT subitem 8b? *

Copy and paste relevant sections from the manuscript (include quotes in quotation marks "like this" to indicate direct quotes from your manuscript), or elaborate on this item by providing additional information not in the ms, or briefly explain why the item is not applicable/relevant for your study

Procedure
Overview
This study was designed as a parallel-design RCT. Randomization was computerized independently by research staff using a blocked randomization scheme (block size 10). Participants were expected to be randomized in a ratio of 1:1 to the intervention or waitlist control group. This study was an open-label RCT as it was not possible to blind the allocation.

9) Mechanism used to implement the random allocation sequence (such as sequentially numbered containers), describing any steps taken to conceal the sequence until interventions were assigned

Does your paper address CONSORT subitem 9? *

Copy and paste relevant sections from the manuscript (include quotes in quotation marks "like this" to indicate direct quotes from your manuscript), or elaborate on this item by providing additional information not in the ms, or briefly explain why the item is not applicable/relevant for your study

We only communicated by email which group each person was assigned to.

10) Who generated the random allocation sequence, who enrolled participants, and who assigned participants to interventions

Does your paper address CONSORT subitem 10? *

Copy and paste relevant sections from the manuscript (include quotes in quotation marks "like this" to indicate direct quotes from your manuscript), or elaborate on this item by providing additional information not in the ms, or briefly explain why the item is not applicable/relevant for your study

Randomization was computerized independently by research staff using a blocked randomization scheme (block size 10).

11a) If done, who was blinded after assignment to interventions (for example, participants, care providers, those assessing outcomes) and how
NPT: Whether or not administering co-interventions were blinded to group assignment

11a-i) Specify who was blinded, and who wasn't

Specify who was blinded, and who wasn't. Usually, in web-based trials it is not possible to blind the participants [1, 3] (this should be clearly acknowledged), but it may be possible to blind outcome assessors, those doing data analysis or those administering co-interventions (if any).

	1	2	3	4	5	
subitem not at all important	○	○	○	○	○	essential

Does your paper address subitem 11a-i? *

Copy and paste relevant sections from the manuscript (include quotes in quotation marks "like this" to indicate direct quotes from your manuscript), or elaborate on this item by providing additional information not in the ms, or briefly explain why the item is not applicable/relevant for your study

No one was blinded after assignment to interventions.

11a-ii) Discuss e.g., whether participants knew which intervention was the "intervention of interest" and which one was the "comparator"

Informed consent procedures (4a-ii) can create biases and certain expectations - discuss e.g., whether participants knew which intervention was the "intervention of interest" and which one was the "comparator".

	1	2	3	4	5	
subitem not at all important	○	○	○	○	○	essential

Does your paper address subitem 11a-ii?

Copy and paste relevant sections from the manuscript (include quotes in quotation marks "like this" to indicate direct quotes from your manuscript), or elaborate on this item by providing additional information not in the ms, or briefly explain why the item is not applicable/relevant for your study

回答を入力

11b) If relevant, description of the similarity of interventions

(this item is usually not relevant for ehealth trials as it refers to similarity of a placebo or sham intervention to a active medication/intervention)

Does your paper address CONSORT subitem 11b? *

Copy and paste relevant sections from the manuscript (include quotes in quotation marks "like this" to indicate direct quotes from your manuscript), or elaborate on this item by providing additional information not in the ms, or briefly explain why the item is not applicable/relevant for your study

The content included “meditation of breath,” “meditation of breath, sound, and body,” and “body scan meditation,” based on previous studies [22]. As the “body scan” was partially included in “meditation of breath, sound, and body,” in this study, the latter was conducted after the former. Furthermore, as the effectiveness of interventions that incorporated elements of self-compassion was recently highlighted, “loving-kindness meditation” was ultimately added. As a daily 13-minute meditation was effective after 8 weeks [23], the intervention period was designed to be 8 weeks.

12a) Statistical methods used to compare groups for primary and secondary outcomes

NPT: When applicable, details of whether and how the clustering by care providers or centers was addressed

Does your paper address CONSORT subitem 12a? *

Copy and paste relevant sections from the manuscript (include quotes in quotation marks "like this" to indicate direct quotes from your manuscript), or elaborate on this item by providing additional information not in the ms, or briefly explain why the item is not applicable/relevant for your study

Statistical Analysis
We conducted Chi-squared, t tests, and the Fisher exact test in order to examine whether there are differences in demographic variables and psychological indices between the intervention and control groups. Subsequently, we conducted 2-tailed t tests to examine whether there were differences in demographic variables and psychological indices of participants in the intervention and waitlist control groups, respectively.
For the intervention effects, we conducted an analysis of covariance (ANCOVA; independent variables: intervention group=1 and waitlist control group=0) that used the least squares method as an estimation method, controlled for preintervention scores. We conducted an ANCOVA that used the least squares estimation method, controlled for preintervention scores, age, employment status (regular employment: employed full time with no fixed term of employment; nonregular employment: not regular employment), psychiatric history, education, and marital status. In this study, the participants were randomly assigned to the intervention and control groups. However, because of the possibility that the intervention effect might not be properly detected due to group differences in preintervention scores and demographic data, we controlled for them. Additionally, paired t tests were conducted to determine any differences in the pre- and postintervention assessments within each group.
An intention-to-treatment analysis was used. R (version 4.3.2; R Foundation for Statistical Computing) was used for statistical analysis.

12a-i) Imputation techniques to deal with attrition / missing values

Imputation techniques to deal with attrition / missing values: Not all participants will use the intervention/comparator as intended and attrition is typically high in ehealth trials. Specify how participants who did not use the application or dropped out from the trial were treated in the statistical analysis (a complete case analysis is strongly discouraged, and simple imputation techniques such as LOCF may also be problematic [4]).

	1	2	3	4	5	
subitem not at all important	○	○	○	○	○	essential

Does your paper address subitem 12a-i? *

Copy and paste relevant sections from the manuscript (include quotes in quotation marks "like this" to indicate direct quotes from your manuscript), or elaborate on this item by providing additional information not in the ms, or briefly explain why the item is not applicable/relevant for your study

An intention-to-treatment analysis was used. R (version 4.3.2; R Foundation for Statistical Computing) was used for statistical analysis.We only used complete data with no missing values.

12b) Methods for additional analyses, such as subgroup analyses and adjusted analyses

Does your paper address CONSORT subitem 12b? *

Copy and paste relevant sections from the manuscript (include quotes in quotation marks "like this" to indicate direct quotes from your manuscript), or elaborate on this item by providing additional information not in the ms, or briefly explain why the item is not applicable/relevant for your study

We do not conducted additional analyses.

X26) REB/IRB Approval and Ethical Considerations [recommended as subheading under "Methods"] (not a CONSORT item)

X26-i) Comment on ethics committee approval

	1	2	3	4	5	
subitem not at all important	○	○	○	○	○	essential

Does your paper address subitem X26-i?

Copy and paste relevant sections from the manuscript (include quotes in quotation marks "like this" to indicate direct quotes from your manuscript), or elaborate on this item by providing additional information not in the ms, or briefly explain why the item is not applicable/relevant for your study

回答を入力

x26-ii) Outline informed consent procedures

Outline informed consent procedures e.g., if consent was obtained offline or online (how? Checkbox, etc.?), and what information was provided (see 4a-ii). See [6] for some items to be included in informed consent documents.

	1	2	3	4	5	
subitem not at all important	○	○	○	○	○	essential

Does your paper address subitem X26-ii?

Copy and paste relevant sections from the manuscript (include quotes in quotation marks "like this" to indicate direct quotes from your manuscript), or elaborate on this item by providing additional information not in the ms, or briefly explain why the item is not applicable/relevant for your study

回答を入力

X26-iii) Safety and security procedures

Safety and security procedures, incl. privacy considerations, and any steps taken to reduce the likelihood or detection of harm (e.g., education and training, availability of a hotline)

	1	2	3	4	5	
subitem not at all important	○	○	○	○	○	essential

Does your paper address subitem X26-iii?

Copy and paste relevant sections from the manuscript (include quotes in quotation marks "like this" to indicate direct quotes from your manuscript), or elaborate on this item by providing additional information not in the ms, or briefly explain why the item is not applicable/relevant for your study

回答を入力

RESULTS

13a) For each group, the numbers of participants who were randomly assigned, received intended treatment, and were analysed for the primary outcome

NPT: The number of care providers or centers performing the intervention in each group and the number of patients treated by each care provider in each center

Does your paper address CONSORT subitem 13a? *

Copy and paste relevant sections from the manuscript (include quotes in quotation marks "like this" to indicate direct quotes from your manuscript), or elaborate on this item by providing additional information not in the ms, or briefly explain why the item is not applicable/relevant for your study

Therefore, of the 215 participants who were randomized, data from 209 participants (intervention group, n=105; waitlist control group, n=104) were finally analyzed, excluding 2 participants who declined to participate in this study and 4 participants who worked <19 hours per week on average in the preintervention assessment.

13b) For each group, losses and exclusions after randomisation, together with reasons

Does your paper address CONSORT subitem 13b? (NOTE: Preferably, this is shown in a CONSORT flow diagram) *

Copy and paste relevant sections from the manuscript (include quotes in quotation marks "like this" to indicate direct quotes from your manuscript), or elaborate on this item by providing additional information not in the ms, or briefly explain why the item is not applicable/relevant for your study

Methods
Participants
A power analysis was conducted to determine the sample size needed for this study (significance=.05; statistical power=.8; effect size=0.4), and a sample size of 100 participants per group, for a total of 200 participants, was needed. The effect size demonstrated in the meta-analysis of the effects of online mindfulness-based interventions on mental health was used as reference (depression Hedges g=0.34; stress Hedges g=0.44) [21].
This study recruited 397 women workers via various media sources, such as crowdsourcing sites and social networking services. Inclusion criteria included those who were (1) biologically female, (2) employed for at least 20 hours per week,(3) owned an iPhone (for convenience of the app used), and (4) aged 18-64 years. Exclusion criteria included those who (1) received treatment for a mental disorder, (2) scored ≥13 on the 6-item Kessler Psychological Distress Scale (K6) Japanese version, (3) were on leave, and (4) were currently pregnant or likely to become pregnant within six months. Among the participants, 95 did not meet the inclusion and exclusion criteria. Hence, 302 women workers who met the criteria were asked to respond to the preintervention assessment, and 215 who completed the assessment were randomly assigned to the intervention (n=107) or waitlist control group (n=108). Randomization was computerized using a blocked randomization scheme (block size 10). A total of 8 working women dropped out. Of the 8 participants, 2 participants (intervention group, n=1; wait-list control group, n=1) declined to participate in this study, 2 participants in the intervention group opted out of the intervention, and 4 participants (intervention group, n=2; wait-list control group, n=2) could not be contacted. After 8 weeks, the participants were asked to respond to the postintervention assessment, and 196 women workers completed the assessment (intervention group, n=95; waitlist control group, n=101). Of 215 participants who completed the preintervention assessment, 4 who worked <19 hours per week on average in the preintervention assessment were excluded from analysis (intervention group, n=1; waitlist control group, n=3). Therefore, of the 215 participants who were randomized, data from 209 participants (intervention group, n=105; waitlist control group, n=104) were finally analyzed, excluding 2 participants who declined to participate in this study and 4 participants who worked <19 hours per week on average in the preintervention assessment.

13b-i) Attrition diagram

Strongly recommended: An attrition diagram (e.g., proportion of participants still logging in or using the intervention/comparator in each group plotted over time, similar to a survival curve) or other figures or tables demonstrating usage/dose/engagement.

	1	2	3	4	5	
subitem not at all important	○	○	○	○	○	essential

Does your paper address subitem 13b-i?

Copy and paste relevant sections from the manuscript or cite the figure number if applicable (include quotes in quotation marks "like this" to indicate direct quotes from your manuscript), or elaborate on this item by providing additional information not in the ms, or briefly explain why the item is not applicable/relevant for your study

回答を入力

14a) Dates defining the periods of recruitment and follow-up

Does your paper address CONSORT subitem 14a? *

Copy and paste relevant sections from the manuscript (include quotes in quotation marks "like this" to indicate direct quotes from your manuscript), or elaborate on this item by providing additional information not in the ms, or briefly explain why the item is not applicable/relevant for your study

Procedure
Overview
This study was designed as a parallel-design RCT. Randomization was computerized independently by research staff using a blocked randomization scheme (block size 10). Participants were expected to be randomized in a ratio of 1:1 to the intervention or waitlist control group. This study was an open-label RCT as it was not possible to blind the allocation.
This study was conducted from July 2023 to January 2024 via web forms. Participants in the intervention group installed the app for meditation after the preintervention assessment. Participants practiced guided mindfulness meditation via the app on their cell phones every day at their convenience for 8 weeks. After 8 weeks, the participants received the postintervention questionnaire via the app and email. The participants in the waitlist control group lived as usual for 8 weeks after the preintervention assessment. After 8 weeks, they also responded to the postintervention questionnaire via email.

14a-i) Indicate if critical “secular events” fell into the study period

Indicate if critical “secular events” fell into the study period, e.g., significant changes in Internet resources available or “changes in computer hardware or Internet delivery resources”

	1	2	3	4	5	
subitem not at all important	○	○	○	○	○	essential

Does your paper address subitem 14a-i?

Copy and paste relevant sections from the manuscript (include quotes in quotation marks "like this" to indicate direct quotes from your manuscript), or elaborate on this item by providing additional information not in the ms, or briefly explain why the item is not applicable/relevant for your study

回答を入力

14b) Why the trial ended or was stopped (early)

Does your paper address CONSORT subitem 14b? *

Copy and paste relevant sections from the manuscript (include quotes in quotation marks "like this" to indicate direct quotes from your manuscript), or elaborate on this item by providing additional information not in the ms, or briefly explain why the item is not applicable/relevant for your study

If a participant expressed a desire to discontinue participation in the study, the study was discontinued.

15) A table showing baseline demographic and clinical characteristics for each group

NPT: When applicable, a description of care providers (case volume, qualification, expertise, etc.) and centers (volume) in each group

Does your paper address CONSORT subitem 15? *

Copy and paste relevant sections from the manuscript (include quotes in quotation marks "like this" to indicate direct quotes from your manuscript), or elaborate on this item by providing additional information not in the ms, or briefly explain why the item is not applicable/relevant for your study

"Results
Baseline
Figure 3 illustrates the CONSORT (Consolidated Standards for Reporting Trials) flow diagram (the CONSORT checklist is provided in Multimedia Appendix 1).
Table 2 shows the participants' demographic characteristics. Chi-squared and t tests revealed no differences in demographic variables and psychological indices between the intervention and waitlist control groups (P>.05)."

"Outcomes
Group Effects
Table 3 presents the scores of the pre- and postintervention assessments. Table 4 presents the results of ANCOVA. The ANCOVA, controlled for preintervention scores, revealed significant group effects on life satisfaction (b=1.47, β=0.11; P=.005), perceived stress (b=−2.00, β=−0.17; P=.01), depressive and anxiety symptoms (b=−1.24, β=−0.15; P=.02), and T-Ang/R(b=−0.59, β=−0.11; P=.04). The ANCOVA, controlled for pre-intervention scores and demographic data (age, employment status, psychiatric history, education, marital status), revealed significant group effects on life satisfaction (b=1.35, β=0.10; P=.02),perceivedstress(b=−1.91,β=−0.16;P=.02),depressive and anxiety symptoms (b=−1.13, β=−0.13; P=.03), and T-Ang/R (b=−0.71, β=−0.13; P=.02).

15-i) Report demographics associated with digital divide issues

In ehealth trials it is particularly important to report demographics associated with digital divide issues, such as age, education, gender, social-economic status, computer/Internet/ehealth literacy of the participants, if known.

	1	2	3	4	5	
subitem not at all important	○	○	○	○	○	essential

Does your paper address subitem 15-i? *

Copy and paste relevant sections from the manuscript (include quotes in quotation marks "like this" to indicate direct quotes from your manuscript), or elaborate on this item by providing additional information not in the ms, or briefly explain why the item is not applicable/relevant for your study

回答を入力

16) For each group, number of participants (denominator) included in each analysis and whether the analysis was by original assigned groups

16-i) Report multiple “denominators” and provide definitions

Report multiple “denominators” and provide definitions: Report N’s (and effect sizes) “across a range of study participation [and use] thresholds” [1], e.g., N exposed, N consented, N used more than x times, N used more than y weeks, N participants “used” the intervention/comparator at specific pre-defined time points of interest (in absolute and relative numbers per group). Always clearly define “use” of the intervention.

	1	2	3	4	5	
subitem not at all important	○	○	○	○	○	essential

Does your paper address subitem 16-i? *

Copy and paste relevant sections from the manuscript (include quotes in quotation marks "like this" to indicate direct quotes from your manuscript), or elaborate on this item by providing additional information not in the ms, or briefly explain why the item is not applicable/relevant for your study

"Results
Baseline
Figure 3 illustrates the CONSORT (Consolidated Standards for Reporting Trials) flow diagram (the CONSORT checklist is provided in Multimedia Appendix 1).
Table 2 shows the participants' demographic characteristics. Chi-squared and t tests revealed no differences in demographic variables and psychological indices between the intervention and waitlist control groups (P>.05)."

"Practice Frequency
Participants in the intervention group used the app for a mean of 42.32 days (75.57%, SD 15.63) in 8 weeks."

16-ii) Primary analysis should be intent-to-treat

Primary analysis should be intent-to-treat, secondary analyses could include comparing only "users", with the appropriate caveats that this is no longer a randomized sample (see 18-i).

	1	2	3	4	5	
subitem not at all important	○	○	○	○	○	essential

Does your paper address subitem 16-ii?

Copy and paste relevant sections from the manuscript (include quotes in quotation marks "like this" to indicate direct quotes from your manuscript), or elaborate on this item by providing additional information not in the ms, or briefly explain why the item is not applicable/relevant for your study

回答を入力

17a) For each primary and secondary outcome, results for each group, and the estimated effect size and its precision (such as 95% confidence interval)

Does your paper address CONSORT subitem 17a? *

Copy and paste relevant sections from the manuscript (include quotes in quotation marks "like this" to indicate direct quotes from your manuscript), or elaborate on this item by providing additional information not in the ms, or briefly explain why the item is not applicable/relevant for your study

The results section provides the results for each group for each primary outcome, the estimated effect size, and its precision.

17a-i) Presentation of process outcomes such as metrics of use and intensity of use

In addition to primary/secondary (clinical) outcomes, the presentation of process outcomes such as metrics of use and intensity of use (dose, exposure) and their operational definitions is critical. This does not only refer to metrics of attrition (13-b) (often a binary variable), but also to more continuous exposure metrics such as “average session length”. These must be accompanied by a technical description how a metric like a “session” is defined (e.g., timeout after idle time) [1] (report under item 6a).

	1	2	3	4	5	
subitem not at all important	○	○	○	○	○	essential

Does your paper address subitem 17a-i?

Copy and paste relevant sections from the manuscript (include quotes in quotation marks "like this" to indicate direct quotes from your manuscript), or elaborate on this item by providing additional information not in the ms, or briefly explain why the item is not applicable/relevant for your study

回答を入力

17b) For binary outcomes, presentation of both absolute and relative effect sizes is recommended

Does your paper address CONSORT subitem 17b? *

Copy and paste relevant sections from the manuscript (include quotes in quotation marks "like this" to indicate direct quotes from your manuscript), or elaborate on this item by providing additional information not in the ms, or briefly explain why the item is not applicable/relevant for your study

We do not use binary outcomes.

18) Results of any other analyses performed, including subgroup analyses and adjusted analyses, distinguishing pre-specified from exploratory

Does your paper address CONSORT subitem 18? *

Copy and paste relevant sections from the manuscript (include quotes in quotation marks "like this" to indicate direct quotes from your manuscript), or elaborate on this item by providing additional information not in the ms, or briefly explain why the item is not applicable/relevant for your study

We do not conduct analyses other than those specified in advance.

18-i) Subgroup analysis of comparing only users

A subgroup analysis of comparing only users is not uncommon in ehealth trials, but if done, it must be stressed that this is a self-selected sample and no longer an unbiased sample from a randomized trial (see 16-iii).

	1	2	3	4	5	
subitem not at all important	○	○	○	○	○	essential

Does your paper address subitem 18-i?

Copy and paste relevant sections from the manuscript (include quotes in quotation marks "like this" to indicate direct quotes from your manuscript), or elaborate on this item by providing additional information not in the ms, or briefly explain why the item is not applicable/relevant for your study

回答を入力

19) All important harms or unintended effects in each group
(for specific guidance see CONSORT for harms)

Does your paper address CONSORT subitem 19? *

Copy and paste relevant sections from the manuscript (include quotes in quotation marks "like this" to indicate direct quotes from your manuscript), or elaborate on this item by providing additional information not in the ms, or briefly explain why the item is not applicable/relevant for your study

Perceived stress improved after 8 weeks in the control group that did not receive the intervention.

19-i) Include privacy breaches, technical problems

Include privacy breaches, technical problems. This does not only include physical “harm” to participants, but also incidents such as perceived or real privacy breaches [1], technical problems, and other unexpected/unintended incidents. “Unintended effects” also includes unintended positive effects [2].

	1	2	3	4	5	
subitem not at all important	○	○	○	○	○	essential

Does your paper address subitem 19-i?

Copy and paste relevant sections from the manuscript (include quotes in quotation marks "like this" to indicate direct quotes from your manuscript), or elaborate on this item by providing additional information not in the ms, or briefly explain why the item is not applicable/relevant for your study

回答を入力

19-ii) Include qualitative feedback from participants or observations from staff/researchers

Include qualitative feedback from participants or observations from staff/researchers, if available, on strengths and shortcomings of the application, especially if they point to unintended/unexpected effects or uses. This includes (if available) reasons for why people did or did not use the application as intended by the developers.

	1	2	3	4	5	
subitem not at all important	○	○	○	○	○	essential

Does your paper address subitem 19-ii?

Copy and paste relevant sections from the manuscript (include quotes in quotation marks "like this" to indicate direct quotes from your manuscript), or elaborate on this item by providing additional information not in the ms, or briefly explain why the item is not applicable/relevant for your study

回答を入力

DISCUSSION

22) Interpretation consistent with results, balancing benefits and harms, and considering other relevant evidence

NPT: In addition, take into account the choice of the comparator, lack of or partial blinding, and unequal expertise of care providers or centers in each group

22-i) Restate study questions and summarize the answers suggested by the data, starting with primary outcomes and process outcomes (use)

Restate study questions and summarize the answers suggested by the data, starting with primary outcomes and process outcomes (use).

	1	2	3	4	5	
subitem not at all important	○	○	○	○	○	essential

Does your paper address subitem 22-i? *

Copy and paste relevant sections from the manuscript (include quotes in quotation marks "like this" to indicate direct quotes from your manuscript), or elaborate on this item by providing additional information not in the ms, or briefly explain why the item is not applicable/relevant for your study

Discussion
Principal Findings
This study examined the effectiveness of a mindfulness meditation intervention via a smartphone app among healthy women workers. To our knowledge, this was the first study that examined the effects of the mindfulness meditation intervention via a smartphone app on 4 domains (psychological, work, family, and work-to-family conflict) among women workers. Women workers who received the intervention demonstrated higher postintervention scores on the general psychological indicators (life satisfaction, perceived stress, depressive and anxiety symptoms, and trait anger (reaction) than those in the waitlist control group, controlled for preintervention scores as well as age, employment status, psychiatric history, education, and marital status. However, the intervention was not effective in the other 3 domains (work, family, and work-to-family conflict). In particular, life satisfaction and depression, and anxiety symptoms significantly improved in the intervention group.

22-ii) Highlight unanswered new questions, suggest future research

Highlight unanswered new questions, suggest future research.

	1	2	3	4	5	
subitem not at all important	○	○	○	○	○	essential

Does your paper address subitem 22-ii?

Copy and paste relevant sections from the manuscript (include quotes in quotation marks "like this" to indicate direct quotes from your manuscript), or elaborate on this item by providing additional information not in the ms, or briefly explain why the item is not applicable/relevant for your study

回答を入力

20) Trial limitations, addressing sources of potential bias, imprecision, and, if relevant, multiplicity of analyses

20-i) Typical limitations in ehealth trials

Typical limitations in ehealth trials: Participants in ehealth trials are rarely blinded. Ehealth trials often look at a multiplicity of outcomes, increasing risk for a Type I error. Discuss biases due to non-use of the intervention/usability issues, biases through informed consent procedures, unexpected events.

	1	2	3	4	5	
subitem not at all important	○	○	○	○	○	essential

Does your paper address subitem 20-i? *

Copy and paste relevant sections from the manuscript (include quotes in quotation marks "like this" to indicate direct quotes from your manuscript), or elaborate on this item by providing additional information not in the ms, or briefly explain why the item is not applicable/relevant for your study

Limitations and Future Directions
This study has some limitations: a lack of subgroup analysis, an intervention not designed specifically for the target or context, and problems with generalizability and variability in the intensity of the intervention due to the application and problems with the scales used. First, in this study, subgroup analyses were not conducted to examine the impact of the subjects' traits on intervention effects. The effects of our mindfulness intervention on general psychological, work-related, family-related, and work-to-family conflict indicators may differ based on other factors. Some participants may have benefited from work-related, family-related, or work-to-conflict indicators. Therefore, it is necessary to examine the factors that moderate the effect of mindfulness interventions.
Second, the mindfulness intervention used in this study was not designed as target- and context-specific. Previous studies have developed target- and context-specific mindfulness interventions, such as for the workplace and parenting. Therefore, future studies should be designed specifically for working women, with an aim to increase the effects on work- and family-related indicators.
Third, there are two limitations of using an app for the intervention: the quality of the intervention cannot be assessed, and generalizability is limited due to restrictions on participant conditions. Since a self-help app was used as the intervention in this study, it was not possible to assess how well participants were focused on meditation, which may have resulted in variability in the effectiveness of the intervention. In addition, the limitations of the app used for the intervention limited the participants in this study to iPhone users, which may have biased the sample and limited generalizability. Therefore, future studies should address compliance issues to address these limitations caused by the app without limiting participants to iPhone users.

21) Generalisability (external validity, applicability) of the trial findings

NPT: External validity of the trial findings according to the intervention, comparators, patients, and care providers or centers involved in the trial

21-i) Generalizability to other populations

Generalizability to other populations: In particular, discuss generalizability to a general Internet population, outside of a RCT setting, and general patient population, including applicability of the study results for other organizations

	1	2	3	4	5	
subitem not at all important	○	○	○	○	○	essential

Does your paper address subitem 21-i?

Copy and paste relevant sections from the manuscript (include quotes in quotation marks "like this" to indicate direct quotes from your manuscript), or elaborate on this item by providing additional information not in the ms, or briefly explain why the item is not applicable/relevant for your study

回答を入力

21-ii) Discuss if there were elements in the RCT that would be different in a routine application setting

Discuss if there were elements in the RCT that would be different in a routine application setting (e.g., prompts/reminders, more human involvement, training sessions or other co-interventions) and what impact the omission of these elements could have on use, adoption, or outcomes if the intervention is applied outside of a RCT setting.

	1	2	3	4	5	
subitem not at all important	○	○	○	○	○	essential

Does your paper address subitem 21-ii?

Copy and paste relevant sections from the manuscript (include quotes in quotation marks "like this" to indicate direct quotes from your manuscript), or elaborate on this item by providing additional information not in the ms, or briefly explain why the item is not applicable/relevant for your study

回答を入力

OTHER INFORMATION

23) Registration number and name of trial registry

Does your paper address CONSORT subitem 23? *

Copy and paste relevant sections from the manuscript (include quotes in quotation marks "like this" to indicate direct quotes from your manuscript), or elaborate on this item by providing additional information not in the ms, or briefly explain why the item is not applicable/relevant for your study

Trial Registration: University Hospital Medical Information Network Clinical Trials Registry (UMIN-CTR) UMIN000051796; https://center6.umin.ac.jp/cgi-open-bin/ctr_e/ctr_view.cgi?recptno=R000059110

24) Where the full trial protocol can be accessed, if available

Does your paper address CONSORT subitem 24? *

Cite a Multimedia Appendix, other reference, or copy and paste relevant sections from the manuscript (include quotes in quotation marks "like this" to indicate direct quotes from your manuscript), or elaborate on this item by providing additional information not in the ms, or briefly explain why the item is not applicable/relevant for your study

We do not have the trial protocol.

25) Sources of funding and other support (such as supply of drugs), role of funders

Does your paper address CONSORT subitem 25? *

Copy and paste relevant sections from the manuscript (include quotes in quotation marks "like this" to indicate direct quotes from your manuscript), or elaborate on this item by providing additional information not in the ms, or briefly explain why the item is not applicable/relevant for your study

Funding
This work was supported by the Japan Science and Technology Agency SPRING (JPMJSP2108 to RU, KA, and MS), the Japan Society for the Promotion of Science (JSPS) Grant-in-Aid for Scientific Research (JP16H05653, JP19K03278, 22H01091, and 22K18582 to RT), the Royal Society and the British Academy (AL150003 to RT), and the University of Tokyo Social Cooperation Program “Fulfillment through Work” (to RT). The funders had no role in data collection or analyses, the decision to publish, or preparation of the manuscript.

X27) Conflicts of Interest (not a CONSORT item)

X27-i) State the relation of the study team towards the system being evaluated

In addition to the usual declaration of interests (financial or otherwise), also state the relation of the study team towards the system being evaluated, i.e., state if the authors/evaluators are distinct from or identical with the developers/sponsors of the intervention.

	1	2	3	4	5	
subitem not at all important	○	○	○	○	○	essential

Does your paper address subitem X27-i?

Copy and paste relevant sections from the manuscript (include quotes in quotation marks "like this" to indicate direct quotes from your manuscript), or elaborate on this item by providing additional information not in the ms, or briefly explain why the item is not applicable/relevant for your study

回答を入力

About the CONSORT EHEALTH checklist

As a result of using this checklist, did you make changes in your manuscript? *

- ○ yes, major changes
- ○ yes, minor changes
- ◉ no

What were the most important changes you made as a result of using this checklist?

回答を入力

How much time did you spend on going through the checklist INCLUDING making changes in your manuscript *

It takes 1 hour to go through the checklist.

As a result of using this checklist, do you think your manuscript has improved? *

- ○ yes
- ◉ no
- ○ その他:

Would you like to become involved in the CONSORT EHEALTH group?

This would involve for example becoming involved in participating in a workshop and writing an "Explanation and Elaboration" document

- ○ yes
- ◉ no
- ○ その他:

選択を解除

Any other comments or questions on CONSORT EHEALTH

回答を入力

STOP - Save this form as PDF before you click submit

To generate a record that you filled in this form, we recommend to generate a PDF of this page (on a Mac, simply select "print" and then select "print as PDF") before you submit it.

When you submit your (revised) paper to JMIR, please upload the PDF as supplementary file.

Don't worry if some text in the textboxes is cut off, as we still have the complete information in our database. Thank you!

Final step: Click submit !

Click submit so we have your answers in our database!

送信 フォームをクリア

Google フォームでパスワードを送信しないでください。

このコンテンツは Google が作成または承認したものではありません。 - フォームのオーナーに問い合わせる - 利用規約 - プライバシー ポリシー

このフォームが不審だと思われる場合 報告

Google フォーム
